# RNA sequencing and transcriptome arrays analyses show opposing results for alternative splicing in patient derived samples

**DOI:** 10.1186/s12864-017-3819-y

**Published:** 2017-06-06

**Authors:** Petr V. Nazarov, Arnaud Muller, Tony Kaoma, Nathalie Nicot, Cristina Maximo, Philippe Birembaut, Nhan L. Tran, Gunnar Dittmar, Laurent Vallar

**Affiliations:** 1 Proteome and Genome Research Unit, Department of Oncology, Luxembourg Institute of Health, Luxembourg, Luxembourg; 2 INSERM UMRS 903, University of Reims Champagne-Ardenne, Reims, France; 3 Departments of Cancer Biology and Neurosurgery, Mayo Clinic Arizona, Phoenix, USA

**Keywords:** Microarrays, RNA sequencing, Differential expression analysis, Differential exon usage, Splicing

## Abstract

**Background:**

RNA sequencing (RNA-seq) and microarrays are two transcriptomics techniques aimed at the quantification of transcribed genes and their isoforms. Here we compare the latest Affymetrix HTA 2.0 microarray with Illumina 2000 RNA-seq for the analysis of patient samples - normal lung epithelium tissue and squamous cell carcinoma lung tumours. Protein coding mRNAs and long non-coding RNAs (lncRNAs) were included in the study.

**Results:**

Both platforms performed equally well for protein-coding RNAs, however the stochastic variability was higher for the sequencing data than for microarrays. This reduced the number of differentially expressed genes and genes with predictive potential for RNA-seq compared to microarray data. Analysis of this variability revealed a lack of reads for short and low abundant genes; lncRNAs, being shorter and less abundant RNAs, were found especially susceptible to this issue. A major difference between the two platforms was uncovered by analysis of alternatively spliced genes. Investigation of differential exon abundance showed insufficient reads for many exons and exon junctions in RNA-seq while the detection on the array platform was more stable. Nevertheless, we identified 207 genes which undergo alternative splicing and were consistently detected by both techniques.

**Conclusions:**

Despite the fact that the results of gene expression analysis were highly consistent between Human Transcriptome Arrays and RNA-seq platforms, the analysis of alternative splicing produced discordant results. We concluded that modern microarrays can still outperform sequencing for standard analysis of gene expression in terms of reproducibility and cost.

**Electronic supplementary material:**

The online version of this article (doi:10.1186/s12864-017-3819-y) contains supplementary material, which is available to authorized users.

## Background

High throughput RNA sequencing (RNA-seq) opened new horizons for transcriptomic studies and has evolved into a standard tool for biological and medical research. So far it has been employed for a large variety of purposes including estimation of gene expression, identification of non-coding genes, detection of new genomic features and drug discovery. It is well established that RNA-seq has strong advantages over the previously developed high-throughput RNA analysis by microarrays. Since quantification is based on sequence reads it can provide data on the expression of exons and exon junctions and thus of genes and their isoforms at a higher dynamic range than microarrays. The data can be reanalysed in silico for identification and separation of different organisms and the initial mapping can be updated if an improved version of the genome is released. Recently, Finotello and co-workers suggested that due to the high reproducibility of RNA-seq [1] technical replicates may be replaced by biological replicates, improving the analysis of biological gene expression variability [2].

However, despite continuous improvement of library preparation protocols, RNA-seq application has its limitations too, which can lead to biases and overvaluation of the results [3–7]. Sequencing is sensitive to the quantity of transcripts. Abundant mRNAs are overrepresented in RNA-seq libraries, attracting the majority of reads. These mRNAs are evaluated with low stochastic variability between samples and thus have increased chances to be found significant by differential expression analysis (DEA) [3]. At the same time, low abundant transcripts receive few reads, which makes them more susceptible to noise and penalizes their chances to be selected by DEA. The quality of the RNA is another source of error. As over 90% of total RNA in the extracts belongs to ribosomal RNA (rRNA) and only 2% to mRNA, special methods must be used to either enrich mRNA (polyA selection) or reduce rRNA levels [3]. Both methods are widely used, but can substantially affect the results. It was found that polyA selection leads to a 3′-end bias in the distribution of reads [5, 8, 9]. At the same time, rRNA depletion can lead to strong unpredictable changes at exon level [5]. The length of a transcript itself can also influence its detection in RNA-seq experiments. A longer transcript has a higher chance to be present in the library and thus to be considered significant after DEA. This in turn may affect the functional annotation of significant genes [6, 10, 11]. Interestingly, for small non-coding RNAs, such as microRNAs, there are evidences that microarrays can outperform sequencing [12].

Finally, the library size has a significant influence on the quality of the analysis. While several million reads may be enough to quantify highly expressed genes, the correct quantification of low abundant genes and transcripts can require as much as 100–200 M reads [13] due to the large differences in abundance between low and high expressed transcripts (spanning 5–6 orders of magnitude). This fact was also illustrated by different studies aimed at the detection of alternative splicing: some studies were performed with only ~30 M–read libraries [14], while others point out that over 400 M of mapped reads should be used [7].

The performance comparison of RNA-seq with microarrays has already been addressed by many studies (e.g. Table 1 in Perkins et al. [15]). In general, a high level of correlation between the two techniques has been reported with a strong emphasis on the advantages of RNA-seq [2, 16–21]. Of great consequence is the fact that most of the researchers, who claim a strong performance advantage of RNA-seq over microarrays, used older versions of these arrays, mainly focused on the abundance of 3′ UTRs, not of entire genes. New array platforms like the Affymetrix Human Transcriptome Arrays 2.0 (HTA) use improved methods for the quantification of transcripts. As the probes in these arrays are evenly spread among all exons and cover exon junctions, they allow estimating the unbiased abundance of a transcript and allow for the analysis of differential exon usage between sample groups. A first comparison was already made by Xu et al. [22] using the previous version of HTA arrays – Glue Grant human transcriptome arrays (GG-H). In this work, it was demonstrated on the reference RNA samples, that these arrays are cost effective, need much less material (50 ng vs 2 μg RNA), show lower between-replicate variability and can detect more significantly expressed genes and exons than RNA-seq with ~46 M uniquely mapped reads. The same group claimed that the overlap of detected alternative spliced exons between microarrays and sequencing was ~50% for the reference samples [23].

**Table 1:** Detection limits and dynamic range of signal and fold change values for RNA-seq and HTA platforms, in log_2_ units

Measure (in log_2_ units)	RNA-seq	HTA
Lower limit of log expression	−0.80	3.83
Higher limit of log expression	9.20	8.89
Dynamic range of log expression	10.00	5.06
Lower limit of absolute logFC	0.67	0.17
Lower limit of absolute logFC	7.55	3.58
Dynamic range of absolute logFC	6.87	3.41

The HTA 2.0 probe sets were redesigned based on the GG-H array and optimized. The new HTA has less redundant probe sets, no SNP-specific probes, updated transcript models and it includes more exon-exon junctions. Importantly, microarrays account now for over 40 k non-protein-coding genes including intergenic RNAs, antisense RNAs and premature miRNAs. The dynamic range of gene expression and log fold-change estimated by the arrays were recently measured on synthetic samples and compared to three other microarray platforms as well as two sequencing techniques [24]. HTA arrays showed promising results in this titration experiment, however the low number of replicates was a limiting factor of this study.

In this work, we compare RNA-seq using a 200 M library to the latest Affymetrix HTA microarrays on tumour and control samples from patients with lung squamous cell carcinoma (SCC). We include here not only the quantification of expressed genes but also the identification of alternatively spliced transcripts that may be implicated in biologically important processes.

## Methods

### Tumour and normal samples

Nine matched pairs of primary tumour and adjacent tissue from lung squamous cell carcinoma patients were collected at the Maison Blanche Hospital, Reims, according to the current EU and French regulations. Upon a careful histological analysis, tissue specimens were stored in liquid nitrogen until use.

### RNA extraction

Total RNA was extracted from biological samples using miRNeasy Mini Kit (Qiagen, Hilden, Germany) according to the manufacturer’s instructions. RNA purity was assessed using a NanoDrop ND-1000 Spectrophotometer (Isogen Life Science) whereas RNA quality was checked using RNA 6000 NanoChips with the Agilent 2100 Bioanalyzer (Agilent, Diegem, Belgium). Only RNA preparations with a RNA integrity number (RIN) >7 were considered for further microarray analysis.

### Transcriptome profiling

#### Human Transcriptome arrays 2.0 (HTA)

100 ng of total RNA was used to process the Affymetrix GeneChip® Human Transcriptome 2.0 Arrays using the GeneChip® WT Plus Reagent Kit according to manufacturer’s instructions (GeneChip® WT PLUS Reagent Kit Manual Target Preparation for GeneChip® Whole Transcript (WT) Expression Arrays P/N 703174 Rev. 2, 2013). The arrays were washed and scanned after 16 h of hybridization.

Quality of Affymetrix HTA microarrays was addressed by Affymetrix spike-in controls, perfect match expression and relative log expression (RLE) during data summarization and normalization in Partek® Genomic Suite.

#### Illumina HiSeq 2000 (RNA-seq)

Preparation of the library for RNA-seq analysis was performed using 1.0 μg of total RNA from each sample and the TruSeq total RNA Sample Preparation Kit version 1.0 (Illumina, San Diego, CA) according to the manufacturer’s instructions. Briefly, total RNAs were fragmented upon depletion of ribosomal RNAs. RNA fragments were used as templates for first-strand cDNA synthesis by reverse transcription with random hexamers. Upon second-strand cDNA synthesis, double-stranded cDNAs were end-repaired and adenylated at the 3′ ends. Following the ligation of universal adapters to cDNA fragments, the sequencing library was generated by PCR, and used to produce the clusters thereafter sequenced on an Illumina HiSeq 2000 (Illumina, San Diego, CA) instrument. Each sample was sequenced in a separate flow cell lane, producing 120–280 M paired-end reads, with a final length of 77 bases.

Microarray and RNA-seq expression data are available at Gene Expression Omnibus under the reference GSE84788.

### Data pre-processing

#### Microarray data pre-processing

The pipeline of data processing for Affymetrix HTA arrays as well as for RNA-seq is illustrated in Additional file 1: Figure S1. Pre-processing of Affymetrix CEL-files was performed with Partek® Genomics Suite version 6.6 (Partek® GS) using the robust multi-chip analysis (RMA) algorithm, which performs background adjustment, quantile normalisation and probe summarisation [25]. GC-content correction was used, as suggested by the default pipeline of Partek® GS. In order to estimate the effect of the normalization procedure, expression data without normalization and with standard RMA normalization (without GC-content correction) were also generated. Further analysis was performed in R/Bioconductor [26].

In order to be able to work with the Ensembl annotation, we matched Affymetrix HTA probe sets to exon coordinates from the human genome release GRCh37.69 (hg19) using the *GenomicRanges* library of R/Bioconductor, and calculated the average expression for each gene and exon. Based on Affymetrix recommendation, junction probe sets were omitted during estimation of gene and exon expression, but were used later for the splicing analysis.

Protein-coding and long non-coding RNAs (lncRNAs) were analysed separately. Several biotypes of genome release GRCh37.69 were combined together in order to cover this species of RNA: “lincRNA”, “antisense”, “processed_transcript”, “sense_intronic”, “sense_overlapping”, “3prime_overlapping_ncrna” and “non_coding”.

#### RNA-seq data pre-processing

Illumina’s pipeline was used to generate the raw FASTQ files, which were then submitted to *TopHat* (v2.0.6) [27]. *Bowtie* (v2.0.2.0) was used as the core read-alignment engine [28]. The mapping was made using default parameters to the reference human genome GRCh37.69 from Ensembl annotation. *TopHat* alignment was able to place 85–95% of the reads from each sample on the human genome (Ensembl GRCh37); the numbers of mapped reads for each sample are given in Additional file 1: Figure S2. Next, aligned BAM files were indexed and sorted with *SAMtools* (v0.1.18.0) for downstream convenience [29].

Counts for gene expression were obtained using *HTSeq* [30]. However, this method cannot be used for exon-level counting due to the high level of exon overlap in the human genome. Exon counting was obtained using the *featureCounts* function of the *Rsubread* R/Bioconductor package, which implements a flexible and powerful counting algorithm [31]. Between-sample normalization at gene level was performed by the R/Bioconductor package *edgeR* [32] using the weighted trimmed mean method (TMM) [33]. Relative scaling factors for the libraries were calculated, and normalized counts per million (CPM) values in *edgeR* were obtained. Additionally, we used fragments per kilobase of transcript per million mapped reads (FPKM) as a measure that should be invariant to the length of genomic features in RNA-seq. FPKM were calculated using the *Cuffdiff 2* algorithm [34]. As this measure is not recommended for differential expression analysis [35], we used it only for correlation analysis.

Exon-level data were normalized within the standard DEXSeq pipeline [36] by the median ratio method [37].

#### Data transformations for exploratory analysis

Exploratory data analysis was performed on log_2_-transformed values of signal intensity. Two measures for gene expression were used. The first measure was log_2_ expression (intensity, counts or CPM) of a genomic feature. Calculated as the mean of log_2_ expression of corresponding probe sets, it is an absolute measure that characterizes each sample independently and allows, to some extent, estimating the quantity of the corresponding mRNA. To avoid infinity after log-transformation of RNA-seq data, a small constant offset was added to the measurements. For CPM, we used 0.5, as proposed by the developers of the *edgeR* package [38], therefore logCPM = log_2_(CPM + 0.5). For FPKM, which can have very small positive values, the constant offset was calculated as 1% percentile of all non-zero values (0.005 in our dataset); therefore logFPKM = log_2_(FPKM + 0.005). The second measure was log_2_ fold change (logFC). It provides a relative expression of a gene or exon in tumour versus near-by normal tissue. When calculated for a pair of samples, it removes the part of the transcription signal common to both tissues for the same patient and allows concentrating on the differences between paired tumour and normal adjacent tissues.

### Exploratory analysis of RNA expression

Principal component analysis (PCA) was used to visualize and investigate the clustering of our data. Features (mRNAs) with no signal in RNA-seq were excluded from consideration to avoid zero variance, expression values from each platform were centred and scaled independently and the standard PCA function implemented in R/Bioconductor (*prcomp*) was applied.

In order to characterize the global inter- and intra-group variability of the data in each platform, we used the principal variance component analysis (PVCA) method, which was developed as a hybrid approach and includes advantages of both PCA and variance component analysis [39]. The method estimates fractions of the total variability which are explained by experimental factors. Unexplained variability can then be used as a measure of data quality, as it should decrease with the reduction of intra-group variability and with the increase of inter-group variability.

Comparison between transcription profiles of samples measured by different techniques was performed by Spearman’s rank correlation which reduces the effect of different scales and influential outliers. Confidence intervals for the mean correlation, calculated over a set of samples, were assigned based on the Student distribution of mean values. When reported, *p*-values for Spearman’s correlations were computed within the *cor.test* function of R/Bioconductor. Mean and standard deviation of gene expression calculated independently for each tissue state and each platform were used to characterize the variability of genes between biological replicates.

### Differential expression analysis (DEA)

Microarray data were analysed by linear models with empirical Bayes statistics from the *limma* package of R/Bioconductor in order to detect differentially expressed genes [40]. For RNA-seq we selected one of the most used methods – *edgeR* [32] – which is based on negative binomial models for counting data. The method was recently reported as adequate for analyses with low number of replicates [41]. We tried both paired analysis, which accounts for patient effect, and unpaired analysis, which assumes no linkage between tumour and normal samples coming from the same patient. As no improvement was seen with paired DEA, we used unpaired analysis for the genes. Benjamini-Hochberg correction was used to control false discovery rate (FDR) among selected significant features (mRNAs or lncRNAs).

We performed DEA on all available features for the two considered types of molecules separately: protein-coding mRNAs and lncRNAs. Significant protein-coding genes identified in this analysis were used afterwards for the functional enrichment analysis. To measure the similarity between the gene lists we used the Jaccard index [42]: (*N*_*HTA*_ ∩ *N*_*RNA-seq*_) / (*N*_*HTA*_ ∪ *N*_*RNA-seq*_), where *N*_*HTA*_, *N*_*RNA-seq*_ are the numbers of the selected features identified by HTA and RNA-seq platforms.

Next, public data for lung squamous cell carcinoma (LUSC RNA-seq v2 dataset) from The Cancer Genome Atlas (TCGA) Research Network (http://cancergenome.nih.gov) was used as a reference dataset. This dataset, generated by highly standardized procedures with thorough quality control, contains RNA-seq data from 51 normal and 502 tumour samples. Due to the high number of samples, around 70% of all genes were found significantly differentially expressed (FDR < 0.01) after *limma* analysis with *voom* correction [38]. Therefore, only the top significant genes were considered as reference. Here, *limma* was used instead of *edgeR* as raw counts were not accessible via the TCGA repository at the time.

### Dynamic range and detection limits

We estimated both dynamic range and detection limits considering only informative features – those which were found significantly differentially expressed between cancer and normal groups (FDR < 0.01). The following conservative approach was used to identify expression borders. For all significant genes we calculated lower and upper signal estimates by taking the 1st and 99th percentile of the average expression. In addition, we quantified the lowest and highest log_2_ fold changes of significant gene expression using the same approach. The 1st percentile of log_2_ fold changes can be used as a signal sensitivity estimation as it characterizes the minimal detectable levels for expression differences in two conditions.

### Predictive capacity evaluation

To find out which technique provides more and better potential marker genes for the classification of tumour and normal tissues, we calculated the areas under the ROC curve (AUC) for each gene measured by both platforms. Calculations were performed using the *caTools* package of R/Bioconductor. The behaviour of potential marker genes was then compared to the markers found in the SCC reference dataset from TCGA.

### Functional analysis

Enrichment analysis on significantly differentially expressed genes was performed using Gene Ontology (GO) terms from all the available domains (biological processes, molecular functions and cellular components) [43]. The main analysis was performed for genes with FDR < 10^−4^ in order to get a manageable number of significant genes, compatible with regular practice of functional annotation, and to level the size of the selected gene lists. However, in order to avoid stochastic effects and artefacts selecting a fixed threshold for the platforms, we verified our findings in parallel with 4 thresholds for FDR (10^−2^, 10^−3^, 10^−4^ and 10^−5^) and 4 thresholds for the number of top significant genes (500, 1000, 2000, 4000). The enrichment analysis was repeated on these lists using the *topGO* package of R/Bioconductor. Enrichment of gene ontology categories with significant genes was quantified by Fisher’s exact test and resulting *p*-values adjusted by Benjamini–Hochberg’s FDR procedure. Next, we summarized the extensive lists of enriched ontology terms by removing redundant terms using REVIGO tools (http://revigo.irb.hr) [44] with the semantic similarity measure “Resnik” and dispensability scores of the categories <0.4.

To avoid a tool-related bias, we confirmed our findings using the *ReactomePA* package of R/Bioconductor [45]. In addition, in order to reduce the transcript length effect on the results of the enrichment analysis, we applied the *goseq* package of R/Bioconductor. This package was developed specifically to address the problem of length-related bias in RNA-seq data when performing functional annotation of significant genes [10].

Finally, we checked the behaviour of lung squamous cell carcinoma oncogenes [46]. These genes are involved in tumorigenesis and can be considered as potential therapeutic targets.

### Analysis of differential exon usage

The analysis of the differential exon usage was performed by R/Bioconductor tools widely used in the field: *diffSplice* method from *limma* for HTA arrays and *DEXSeq* package aimed at RNA-seq data analysis [36]. Both *diffSplice* and *DEXSeq* are based on the splicing index (SI) – the difference between exon and gene logFC – and provide the significance of SI. Both algorithms were used twice for each platform: for expression of exons and for expression of exon-exon junctions. Following the requirements of *DEXSeq*, paired analysis was used for exon level. Resulting *p*-values were corrected by the Benjamini-Hochberg’s method. Note that SI based methods can detect exons with zero logFC when the absolute gene logFC is high. To prevent this scenario, we combined FDR and exon logFC selecting significant exons. The logFC of exon expression was calculated from the normalized data.

Next we investigated the potential sources of bias in the detection of splicing events, namely exon length, the relative location of spliced exons and their GC-content. In order to calculate relative exon locations, we used a straightforward approach. We ordered the exons of each gene from 5′ to 3′ terminus, accounting for the strand, assigned them with ranks (from 1 to the number of exons) and then scaled the ranks by the total number of exons. This resulted in values in the [0,1] range for the exons of each gene.

## Results

### Exploratory analysis of the gene expression data

#### Sequencing shows higher variability in the expression data

We started the gene expression analysis by mapping Affymetrix probe-sets on Ensembl-defined exons. This increased the compatibility between the platforms in terms of the lists of characterized genes, especially for protein coding mRNAs: almost 100% of these genes were found in common between both platforms (Additional file 1: Figure S3). Regarding lncRNAs, 5855 (93%) were also found in HTA data. Overlap of exon IDs was high as well: 92% for protein coding mRNAs and 90% for lncRNAs. Therefore, the list of common genes was considered representative enough and further exploratory analyses, such as principal component, variance and correlation analysis, were performed on this list.

**Fig. 1:**
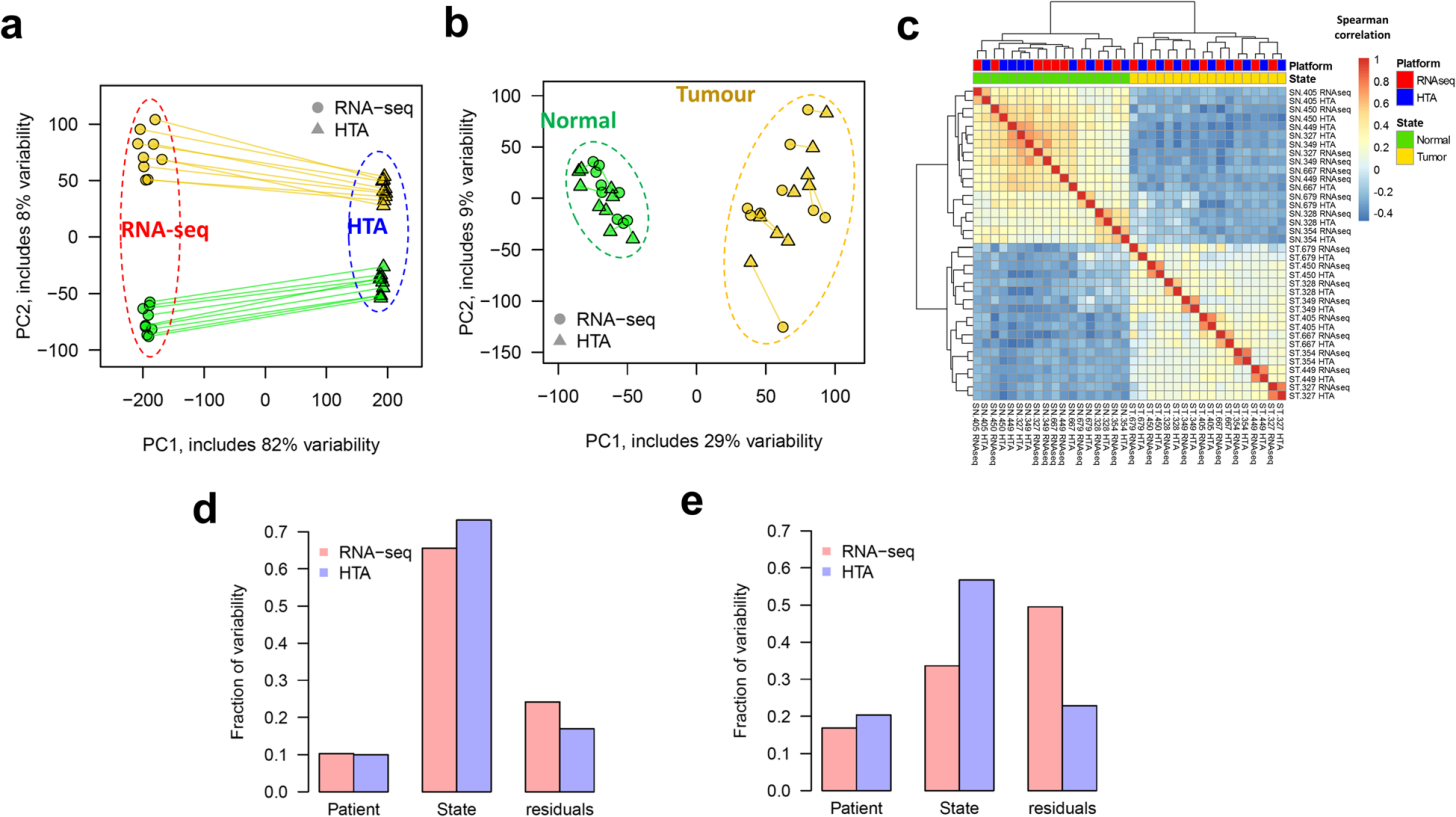
Data variability in two tissue states captured by different platforms: HTA and RNA-seq. PCA of log_2_ expression data for protein coding genes shows clustering based on platform for original data (**a**) and clustering based on tissue state for standardized data (**b**). Lines connect the same samples measured by the two platforms. The heatmap of Spearman correlations between expression profiles measured by both platforms shows that the major difference in gene order is tissue-related, not platform-related (**c**). The fraction of variability, determined by PVCA, is presented for protein-coding genes (**d**) and lncRNAs (**e**). Variability which cannot be explained by patient or tissue state is presented in the “residuals” group

Initially, principal component analysis (PCA) of the expression data showed a strong platform effect (Fig. 1a), which naturally came from the difference in the scale of gene expression offered by each platform. However, a simple linear normalization, such as centring and scaling performed on log-transformed data, strongly reduced this effect (Fig. 1b). Normalized data formed two distinct clusters in the PCA-plot: tumour and normal. As expected, tumour samples demonstrated much higher variability than normal samples, due to the high heterogeneity of tumours between patients. Next, the ranks of the genes in all the samples were compared by Spearman correlation (Fig. 1c). Clearly, the two groups identified by hierarchical clustering, corresponded to the two tissue states: tumour and normal. For tumours, the closest distance was seen for the same samples measured by different platforms.

To further estimate the impact of the experimental factors on mRNA and lncRNA expression data, we applied a principal variance component analysis (PVCA) [39] method which quantified fractions of variability associated with two factors: patient and tumour/normal tissue state. The part of variability that could not be explained by these factors, or “within-group” variability, was represented by residuals. For both types of RNA, the highest fraction of variability detected by HTA arrays was associated with the tissue state (Fig. 1d,e). The patient effect was less pronounced than the tissue effect and comparable between the platforms. Importantly, the highest residuals were observed in RNA-seq data, suggesting higher levels of stochastic noise in this dataset. To prove this, we investigated the variability between biological replicates in normal and tumour tissues with respect to the average expression level (Additional file 1: Figure S4). For both tissues, higher variability was observed in RNA-seq data, especially for low expressed genes (Additional file 1: Figure S4a,d). This is in agreement with the fact that RNA-seq may be insensitive to transcripts of reduced abundance. In microarrays (Additional file 1: Figure S4b,e), the variability was lowest for the least expressed genes and gradually increased up to a certain plateau (around log_2_ expression of 6) (Additional file 1: Figure S4c,f). The same analysis was repeated using the length-normalized FPKM measure for RNA-seq data, and the results strongly supported the conclusions based on CPM (data not shown).

Altogether, these observations suggested that the variability found in the normalized expression data was mainly attributable to the biological state of the samples (normal vs tumour), with a minor bias linked to the platform, and that higher stochastic variability should be expected in RNA-seq compared to HTA, at least under the considered experimental conditions.

#### Effect of data normalization on the correlation between platforms

As a next step, we compared the observed transcription profiles between the two platforms. Two measures were considered: log_2_ expression and log_2_ fold change between tumour and normal samples coming from the same patient. While the first directly reflects the observed signals, the second reduces the patient effect and highlights the differences between tumour and normal tissue transcriptomes. Spearman correlation was calculated between the expression profiles of the platforms for each sample (or patient for logFC). We observed a relatively good consensus between HTA and RNA-seq platforms for protein-coding mRNA: log-expression signals showed a mean correlation (with a 95% confidence interval) of 0.760 ± 0.007, and a logFC of 0.743 ± 0.053 (all *p*-values were below 10^−16^). However, for lncRNAs these correlations were strongly reduced to 0.319 ± 0.008 and 0.349 ± 0.039, respectively. We detected much higher variability in the correlation coefficients calculated for logFC (F-test resulted in *p*-values of 2.5⋅10^−7^ and 2.0⋅10^−4^ for mRNA and lncRNA, respectively). Due to such variability, we did not detect significant differences in the mean correlations between these measures (*p*-values of 0.49 and 0.12).

We also investigated potential effects of microarray data pre-processing (normalization, GC-content and background correction) that could affect the correlation with RNA-seq data. Not normalized HTA data showed a Spearman correlation of 0.580 ± 0.019 and data normalized by classical RMA – a correlation of 0.588 ± 0.019. As mentioned previously, the default analysis was made with GC-correction and resulted in much higher correlation. Thus, GC-correction is an important step that strongly increases the similarity between Affymetrix HTA and Illumina RNA-seq results.

Next, we checked whether a length-corrected measure of gene expression (such as FPKM) could improve the correlation between the platforms. Indeed, FPKM showed a slight, but consistent, improvement in the correlation between platforms: 0.782 ± 0.006 and 0.376 ± 0.007 for mRNAs and lncRNAs, correspondingly. However, logFC measures calculated over FPKM showed significantly lower correlations than corresponding expressions: 0.690 ± 0.045 and 0.317 ± 0.036 for mRNAs and lncRNAs (*p*-values of 0.001 and 0.005).

#### Gene length has a significant influence on detection levels

**Fig. 2:**
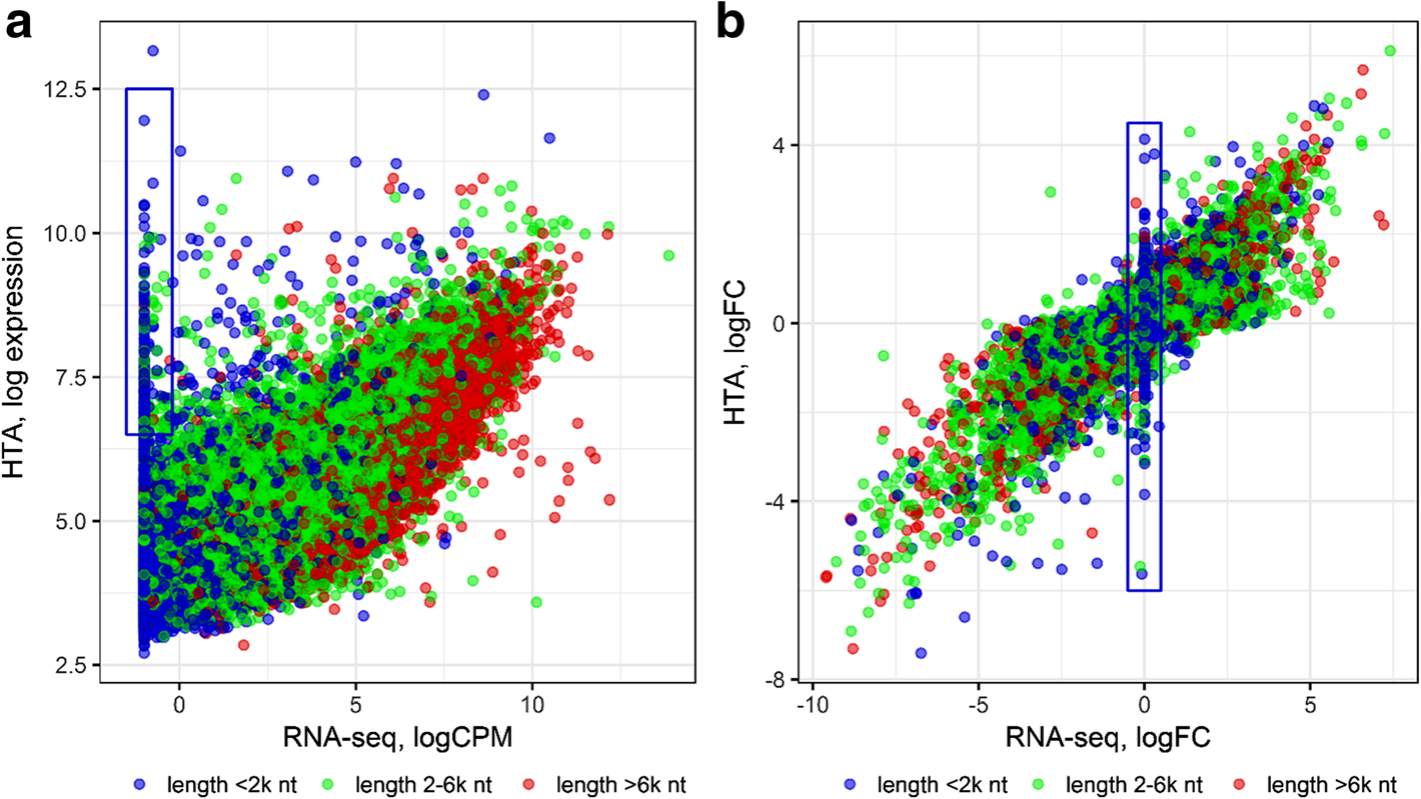
Scatter plots of coding mRNA expression captured by the two platforms. RNA-seq and HTA expression data for one sample (**a**) and logFC for one patient (**b**). Protein coding genes are visualized by gene length: blue – short, green – intermediate, red – long. The blue boxes highlight the genes missed by sequencing, but detected by microarrays

Microarray and sequencing expression profiles were compared using scatter plots of the expression values for each sample (one example is given in Fig. 2a). The range of the detected log_2_ intensity values varied from 3 to 13 in HTA and from −1 to 14 in RNA-seq, which underlines the higher dynamic range of RNA-seq. The plot shows a non-linear relation between the two platforms for low expressed genes. This is probably due to background fluorescence, which starts playing a considerable role in microarrays, when the signal from hybridized transcripts is low. Some protein-coding genes that were not detected by RNA-seq, were captured and showed a moderate signal in HTA (dots within the blue box in Fig. 2a). Partially, this can be explained by the length of the transcripts: short genes (blue dots in Fig. 2) had lower chance to be detected by RNA-seq. At the same time, long genes (red dots) showed highest expression in RNA-seq. The fold change between tumour and normal tissues of a patient (Fig. 2b) depended less on the length of the genes. Nevertheless, a cluster with zero logFC in RNA-seq was mainly formed by short genes.

### Differential expression analysis

**Fig. 3:**
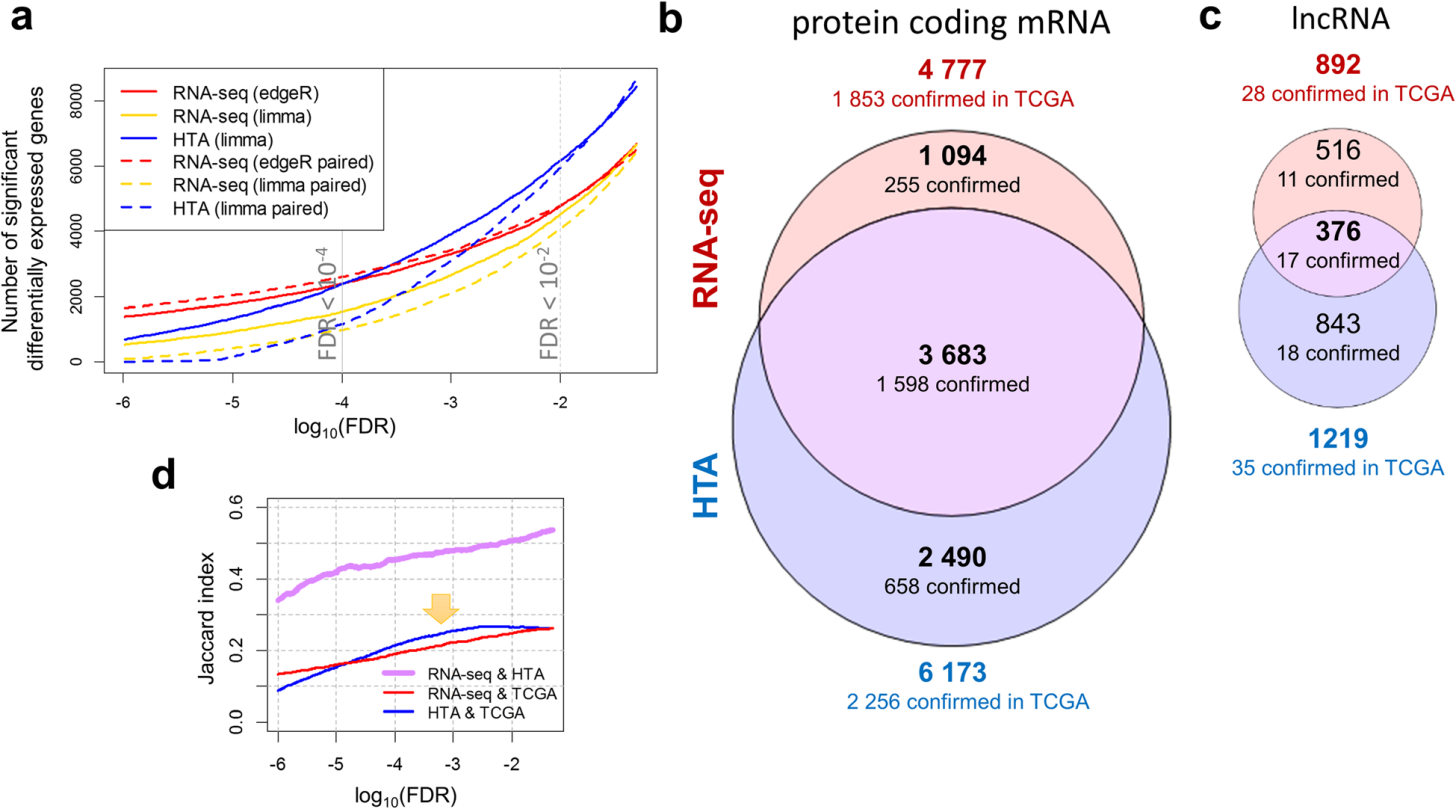
Differentially expressed genes identified by the platforms. Evolution of the number of significant genes identified with variable FDR thresholds (**a**), using *edgeR* and *limma* with *voom* correction for analysis of RNA-seq data, and using *limma* for HTA data. Solid lines show unpaired analyses, while dotted lines show analyses paired by patient. Differentially expressed protein coding mRNAs (**b**) and lncRNAs (**c**) were obtained by unpaired differential expression analysis using *edgeR* for RNA-seq and *limma* for HTA (FDR < 0.01) and represented as proportional Euler-Venn diagrams. The lists of differentially expressed genes were confirmed by the top 25% significant genes detected in the LUSC-TCGA dataset: 4569 protein coding genes (FDR < 10^−18^) and 111 lncRNAs (FDR < 10^−8^) were used. Evolution of Jaccard index for coding mRNAs with variable FDR thresholds (**d**) between the two platforms (violet) shows a monotonic behaviour. Similarity between the TCGA validation gene list and each of the platforms– RNA-seq (red) and HTA (blue) showed a slight outperformance of HTA (marked by an arrow)

In order to investigate how many genes could be found significantly differentially expressed by the platforms, we applied the two most accepted DEA approaches: *limma* and *edgeR*. The first question addressed was whether paired or unpaired analysis should be used. Paired analysis allows for efficient removal of patient-to-patient variability, allowing detection of minor variability linked to the factor of interest, especially when the effect of the treatment is comparable or lower than the patient variability. At the same time, introducing an additional variable into a statistical model can reduce its power and increase the resulting *p*-values, if the patient variability is low. Our analysis of data variability suggested that the patient effect was minor compared to the effect of the tissue state and even lower than the noise level (Fig. 1d). Nevertheless, both paired and unpaired analyses were tested. We observed that pairing of the samples (dotted lines in Fig. 3a) slightly increased the number of significant genes in RNA-seq analysed by *edgeR* but reduced the number of significant genes in HTA analysed by *limma*. In addition, pairing also reduced the list of commonly identified genes. In order to investigate this problem deeper, we repeated the RNA-seq analysis using *limma* with *voom* data transformation [38] (yellow lines in Fig. 3a). Interestingly, *voom/limma* applied to the same RNA-seq data as *edgeR,* showed a behaviour similar to *limma* on HTA, strongly penalizing pairing. Therefore, the effect of pairing was linked to the properties of the statistical model and test used: an algorithm using a specific negative binomial model for gene expression and a likelihood ratio test improved the results (although slightly in our case). *Limma*, which uses a more general normal model for the signal, tends to penalize additional factors strongly. Taking these facts into account we decided to continue the study using the unpaired analysis.

Interestingly, RNA-seq data analysed by *edgeR* reported fewer genes than HTA data analysed by *limma* for moderate FDR values (0.05–10^−4^). For the standard threshold (FDR < 0.01), 6173 protein-coding mRNAs were detected from HTA data while only 4777 were obtained from RNA-seq data, with 3683 genes in common (Jaccard index of 0.507), as shown in Fig. 3b. Likewise, 1219 lncRNAs were found to be significant on the HTA platform against 892 in the RNA-seq experiment (Fig. 3c), but with much lower similarity (0.217). However, genes with a more stringent threshold (FDR < 10^−4^) were observed mainly for *edgeR* with its negative binomial model. When the analysis assumed the normal model, the reported number of significant genes fell even faster with the decrease of FDR for RNA-seq than for HTA arrays (Fig. 3a). As the two curves representing the number of significant genes in HTA and RNA-seq crossed around FDR < 10^−4^ (Fig. 3a), this threshold was used to select the similar-size sets of significant genes for the functional annotation.

In order to verify the obtained lists of differentially expressed genes, we compared them with external results obtained on squamous cell carcinoma samples from TCGA (LUSC dataset). This dataset can be used as a reference due to its high quality and the large size of the patient cohort used. As mentioned in Methods, 25% of the most significant genes in the TCGA dataset were selected – creating a validation gene set of similar size to the set of significant genes in our experiments. For protein-coding mRNAs the validation set was composed of 4569 significant genes with an FDR < 10^−18^. The number of genes identified in our analysis and confirmed by the TCGA dataset are summarized in Fig. 3b,c. HTA showed a higher number of confirmed mRNAs, but proportionally the confirmation level varied only slightly: 37% for HTA, and 39% for RNA-seq. Common mRNAs showed the highest confirmation level (43%), while the uniquely identified genes had lower confirmation rates of 23% and 26% for sequencing and microarrays, respectively. We also investigated the evolution of overlap between platforms and the confirmation rate within each platform (Fig. 3d). Overlap between significant genes detected by both platforms increased with the increase of the FDR threshold. Interestingly, HTA was more similar to TCGA data than RNA-seq for FDR thresholds within the range 10^−5^–10^−2^.

Unlike protein-coding genes, lncRNAs showed very low similarity between our data and the TCGA dataset as only 442 lncRNAs were quantified in TCGA. Therefore, the top 111 were used (FDR < 10^−8^) as a reference list. The confirmation level was only 5% for lncRNAs commonly detected by both platforms.

In summary, the differential expressed analysis showed a higher number of significant genes in HTA compared to RNA-seq (for FDR < 0.01). The genes commonly identified by both platforms exhibited a higher confirmation rate by comparison to the results from the TCGA dataset. The results of the differential expression analysis are provided in Additional file 2.

### Dynamic range and sensitivity

Current literature repeatedly mentions a significant difference in the dynamic range of these two platforms. Here the detection limits and dynamic ranges of the platforms were determined using the significantly differentially expressed protein-coding genes. The lower and upper limits of the average log_2_ expression and fold change are given in Table 1 (in log2 units) and can be visualized in Additional file 1: Figure S5-S6. The lowest detection limit for RNA-seq was −0.8 in logCPM scale (Additional file 1: Figure S5a), with a theoretical minimum for logCPM = −1, as defined by the added constant of 0.5. In our experiments, this corresponded to a detectable increase from 0 raw counts in one condition to 2–4 raw counts per gene (on average) in another condition. HTA expression was shifted to higher values with a minimum around 3.8 due to the background signal always observed in microarrays. As expected, the dynamic range of RNA-seq outperformed the one of HTA (10 vs ~5 log_2_ units). This can also be seen in MA-plots in Additional file 1: Figure S6 (comparing left panels to right ones). These figures suggest that lower expressed genes tend to show higher logFC in RNA-seq, but not in microarrays.

In order to compare the sensitivity in detecting differences of gene expression we also calculated logFC. The dynamic range of logFC changed less dramatically – 6.87 for RNA-seq and 3.4 log_2_ units for HTA (Additional file 1: Figure S5b,d). Of note, the detection limit of logFC was lower for HTA (0.17) than for RNA-seq (0.67). The majority of protein coding genes showed expression above the defined lower detection limits: 81.0 ± 0.7% for RNA-seq and 90.1 ± 0.4% for HTA. For lncRNA, a somewhat smaller proportion was observed: 57.2 ± 2.7% and 63.4 ± 1.5% for RNA-seq and HTA, respectively. As can be noted, higher factions of mRNA and lncRNA were observed as expressed in HTA. Thus, HTA microarrays provided a higher number of genes with small but statistically significant logFCs than sequencing.

### Predictive capacity of expression data

**Fig. 4:**
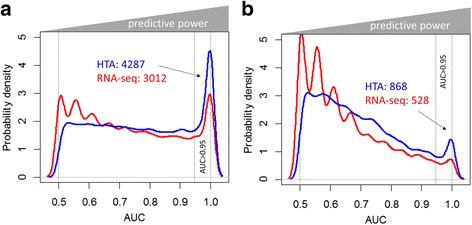
Distribution of AUC values for classification of tumour and normal samples. The red curve corresponds to RNA-seq data and the blue to HTA data for protein coding mRNA (**a**) and lncRNA (**b**). More genes with high AUC, and therefore higher predictive power, were seen for the HTA platform. Fluctuations of RNA-seq distribution for low AUC values are artefacts linked to the limited number of samples

The next step was to investigate which platform provided more genes with predictive capacity in discriminating cancer from normal samples. Calculations were performed as described in Methods. Distributions of AUC values for protein coding mRNAs and lncRNAs are shown in Fig. 4. A higher number of genes with AUC values close to 1, and therefore higher diagnostic capacity, were seen for the HTA platform, for both types of RNA. An AUC > 0.95 was shown by 4287 protein-coding mRNAs in microarray data, while only 3012 genes showed the same predictive capacity in RNA-seq (Fig. 4a). Both platforms were able to detect 2344 mRNAs as markers (Jaccard index of 0.473). As for the DEA, these results were verified with the TCGA dataset, where 2016 genes with AUC > 0.95 were selected as a validation set for predictive markers. Almost the same similarity was observed between the individual marker lists and the TCGA-based list (~0.16). Unlike for DEA, intersection of the markers from the two platforms did not improve the similarity with the TCGA-based markers. The same tendency was observed for lncRNAs: HTA detected more genes with higher predictive capacity. However, a much smaller portion of non-coding genes was found with AUC > 0.95 and both distributions in Fig. 4b show higher density near small AUC values. Nevertheless, 528 markers were identified for RNA-seq and 868 for HTA with slightly higher similarity (0.22).

### Functional annotation of differentially expressed genes

**Fig. 5:**
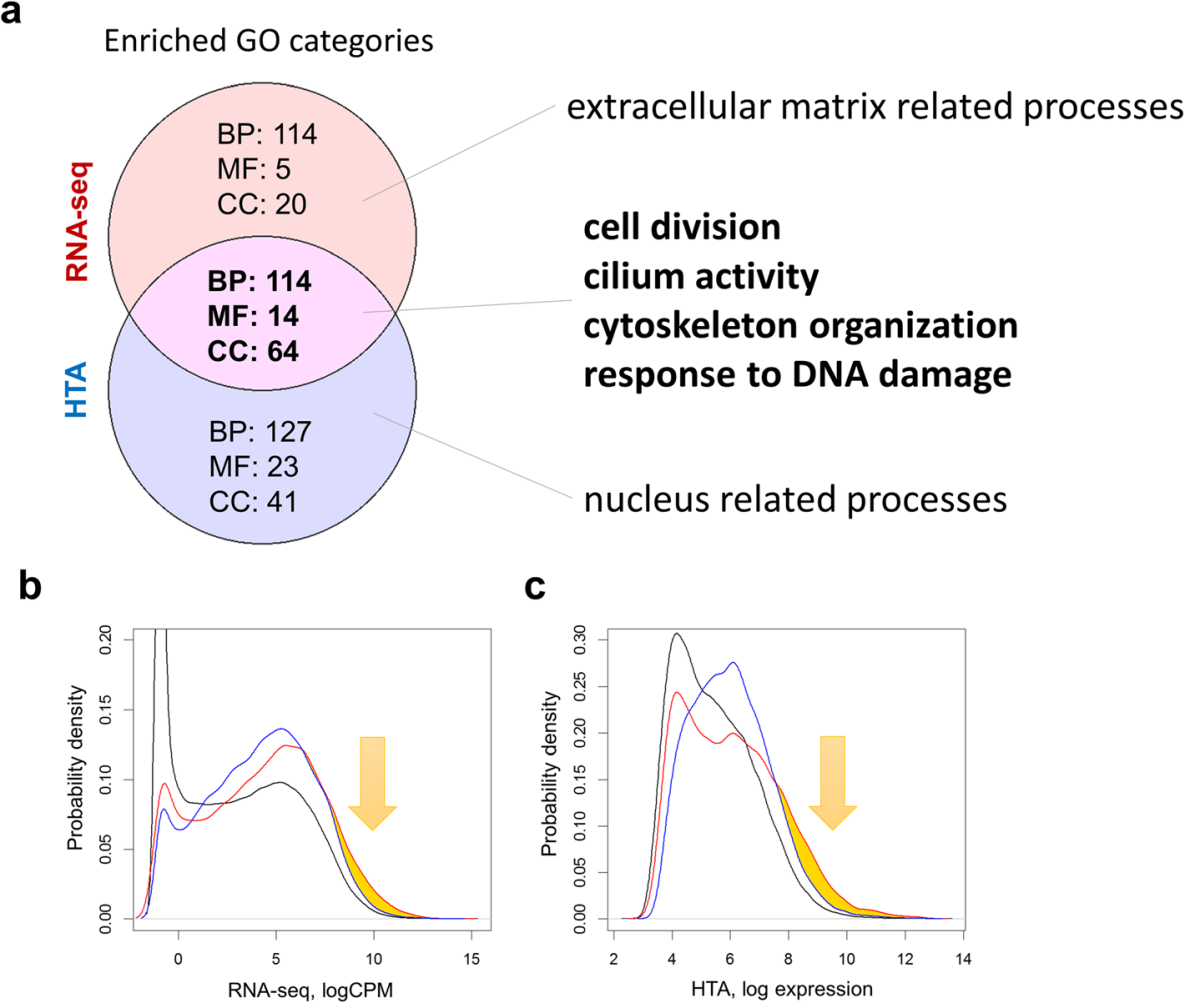
The results of the functional annotation of significant genes. Enriched gene ontology (GO) biological processes (BP), molecular functions (MF) and cellular components (CC) by significant (FDR < 10^−4^) protein coding genes from unpaired RNA-seq (*edgeR*) and HTA (*limma*) analyses are intersected (**a**). Only ontology terms with FDR < 0.01 were considered. The complete list is given in Additional file 3 and summarized in Additional file 1: Figure S7. The expression of genes related to cellular component ontology terms uniquely identified by RNA-seq (*red lines*) or HTA (blue lines) is shown (**b**-**c**). The distributions of gene expressions are based on sequencing (**b**) and microarray (**c**) data. Both analyses show that genes associated with an ontology uniquely found in RNA-seq analysis have a higher expression than genes with HTA-specific ontology terms (arrows and yellow area)

Significantly differentially expressed protein-coding genes were functionally annotated considering gene ontology biological processes, molecular functions and cellular components categories as described in Methods. A stringent FDR < 10^−4^ was used, in order to reduce and adjust the number of significant genes to 2390 genes in RNA-seq and 2382 genes in HTA. More categories were significantly enriched (FDR-adjusted Fisher’s *p*-value <0.01) in the data from the HTA platform: 241 for biological processes, 37 for molecular functions and 105 for cellular components (Fig. 5). RNA-seq data enriched categories included 228 genes for biological processes, 19 for molecular functions and 84 for cellular components terms. The similarity in enriched GO terms was lower than for genes (e.g. Jaccard index of 0.321 for biological processes). By cross-validation (leaving 10% of genes out) we identified, that ontology terms with high FDR and low number of member genes were particularly responsible for this dissimilarity. Enriched terms found in common and specifically by each platform were then combined into generalized categories by REVIGO as mentioned in the Methods section (Additional file 1: Figure S7). Significantly enriched terms are listed in Additional file 3.

Results from both platforms pointed to strong differences between tumour and normal tissues associated with cell cycle-related processes and cilium activity. As expected, genes involved in cilium activity of normal lung epithelium are less expressed in tumour cells. This is in line with the phenotype of degenerated differentiation and unlimited cellular growth in tumours. Other common processes found by the platforms were cell division, microtubule-based movement, DNA reorganization and DNA repair. In contrast to the genes involved in these processes, we identified a clear difference between RNA-seq and HTA results. Among the biological processes (Additional file 1: Figure S7a) a stronger signal was seen in RNA-seq data for tissue development and extracellular matrix organization, while in HTA a stronger change in DNA-related processes was observed. For cellular components (Additional file 1: Figure S7c), extracellular matrix and cell-cell junction were uniquely identified in RNA-seq, while nucleoplasm and intracellular part were mainly observed in HTA data. Despite the fact that only a few molecular functions were found enriched (Additional file 1: Figure S7b), the same tendency was seen with protein binding involved in cell adhesion detected uniquely in RNA-seq, and poly(A)-RNA binding and nucleoside-triphosphatase activity observed exclusively in HTA. In order to explain this discrepancy, the hypothesis that gene length can bias the enrichment results [10] was tested by comparing the distribution of lengths from genes involved in unique GO terms. However, no effect was observed. The enrichment analysis of significant RNA-seq genes was repeated using the *goseq* package, designed to account for gene length variability, but the results supported the findings of *topGO* and did not remove the bias. We also excluded the effect of the background gene list. As suggested in the work of Timmons et al., we repeated the analysis leaving out not- or low expressed genes from the enrichment analysis (both from query and background gene lists) [11]. Only genes with an expression level above the median in at least 50% of the samples were kept. This correction decreased the number of enriched ontology terms in general, but did not remove the bias in the cellular components. Finally, in order to exclude database or tool-specific biases, we repeated the enrichment with the *ReactomePA* tool. The categories found enriched supported the general trend observed for GO: more enriched pathways were observed in the microarray-derived list of significant genes and the similarity between the pathways was 0.298. Top common pathways were linked to cell cycle (FDR < 10^−21^). Top HTA-specific pathways were chromosome maintenance and DNA repair (FDR < 10^−7^), while the top RNA-seq-specific pathway was extracellular matrix organization (FDR < 10^−9^).

The bias found in the enriched biological functions may be linked to the abundance of the transcripts. Indeed, a group of low expressed genes may stay undetected or be detected with high variability. The expression of the genes related to GO terms uniquely found by RNA-seq or HTA was consequently investigated. The distributions of the expression of the genes related to cellular component ontology terms which were uniquely identified by each of the platforms are shown in Fig. 5b,c. Both platforms showed that genes from RNA-seq-specific GO terms (i.e. extracellular region) are expressed at higher levels than those from HTA-unique ontology terms (nucleoplasm). Therefore, the difference in sensitivity to low expressed transcripts may at least partially explain the observed bias in cellular components.

Finally, the expression of known lung squamous cell carcinoma oncogenes [46] was tested. The scatterplot of average logFC measured by the two platforms showed a strong concordance (Additional file 1: Figure S8) between the platforms with a Spearman’s correlation of 0.991 (*p*-value 3.2⋅10^−6^). Some oncogenes showed low absolute fold-change between tumour and normal tissue as a consequence of being affected by mutations at DNA level rather than being differentially expressed.

Thus, both platforms were able to capture crucial activities of cancer cells related to an increased rate of cell division and to a loss of normal functionality of the airway epithelium, such as cilium motility. Oncogenes of lung squamous cell carcinoma also showed concordant behaviour. However, we detected a bias between the platforms: RNA-seq tended to detect more abundant genes, active at the extracellular matrix, while HTA showed more genes active within the nucleus.

### Exon level analysis

Both platforms are capable of analysing alternative splicing: they can measure the abundance of exons and their junctions. We compared their similarity at exon level. Correlation between expression of exons decreased (*r* = 0.658 ± 0.010) compared to gene expression (Fig. 2a) and the scatter plot showed stronger variability between the platforms (Additional file 1: Figure S9a). Between-replicate variability also increased for both platforms (Additional file 1: Figure S9b,c). This is reasonable, as expression of gene level is based on the total number of reads mapped to exons or probes targeting them.

**Fig. 6:**
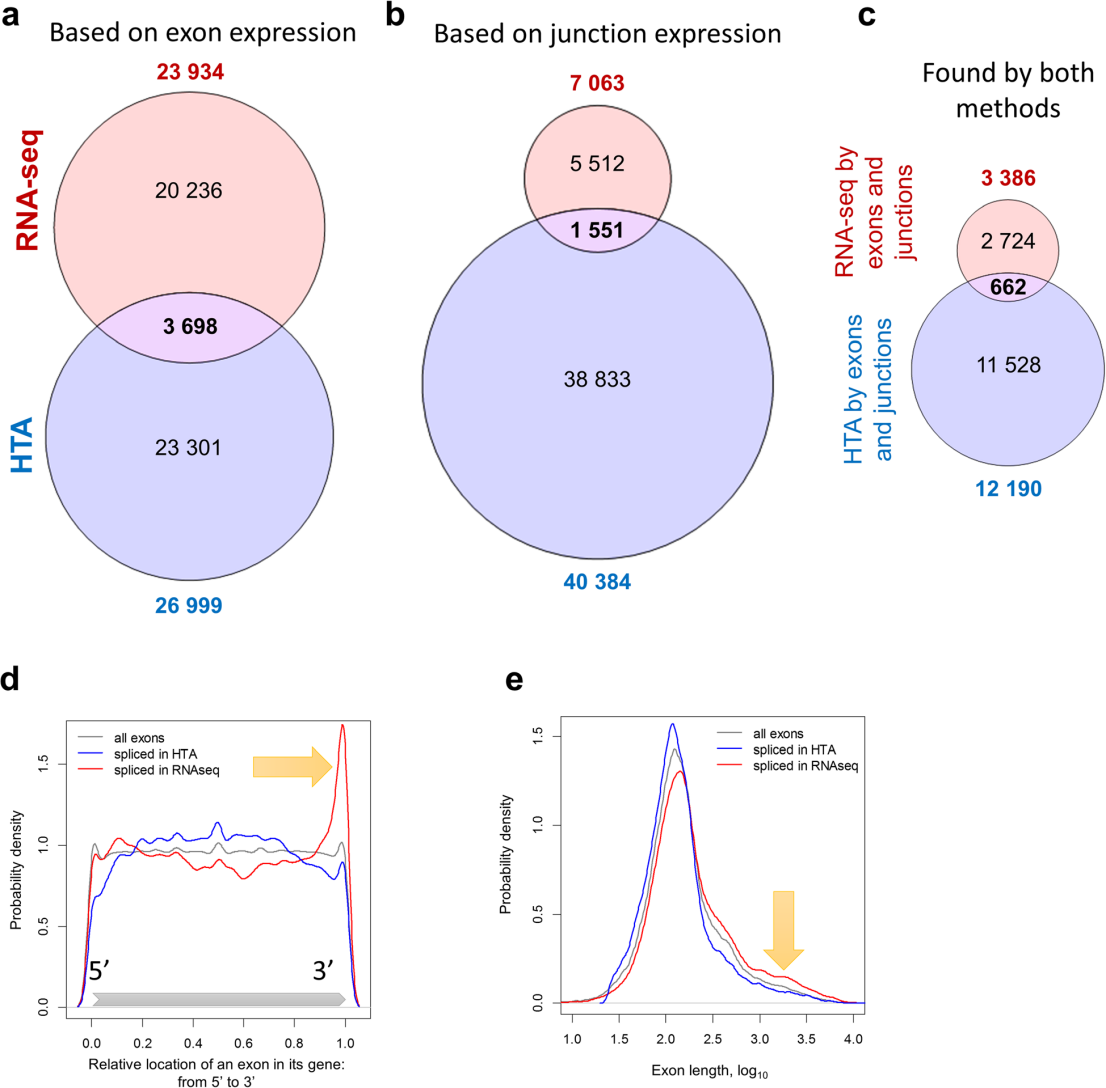
Differential usage of exons detected by RNA-seq and HTA show low similarity. The analysis was based on exon (**a**) or junction (**b**) expression with FDR < 0.05 and |logFC| ≥ log_2_(1.5). The intersection of exons confirmed by both approaches within RNA-seq and HTA platforms is shown (**c**). The exon parameters distribution among differentially used exons detected by the two platforms is also show in (**d**,**e**). The relative position of the exons within their genes, varying from 5′ end (relative position = 0) to 3′ end (relative position = 1), shows a 3′ bias in RNA-seq (**d**). Exon length shows that RNA-seq tends to find more significantly splice events among long exons than HTA (**e**)

The alternative splicing analysis was performed using two approaches as described in Methods. First, differential usage of exons was analysed based on exon expression. Then, differential usage of exon-exon junctions was estimated. Unlike gene-level DEA, differential exon usage analysis returned quite divergent results, as shown in Fig. 6. RNA-seq resulted in 23,934 differentially used exons, with FDR < 0.05 and |logFC| > log_2_(1.5); HTA identified 26,999 alternatively spliced exons. However, only 3698 of these exons were found in common between the two lists (Jaccard index of 0.078). The Spearman rank correlation between FDR values was small (*r* = 0.056) but significantly above zero (*p*-value <10^−16^). An even smaller concordance was observed analysing exon junctions: 7063 junctions were found differentially used by RNA-seq and 40,384 by HTA, with 1551 junctions in common (Jaccard index of 0.034). We identified exons which were detected as spliced by both methods for each platform independently and noticed that within platform similarity was slightly higher (0.107 for RNA-seq and 0.159 for HTA). Comparing the lists of genes with detected differentially used exons we noticed a much higher overlap (Jaccard index of 0.305, considering only exons, and 0.234, considering exons and junctions). However, the majority of exons with splicing events were not concordant.

The lists of exons detected by both exon and junction expression analysis for each platform were intersected in Fig. 6c and resulted in 662 common exons that were reported in Additional file 4. These common differentially used exons belong to 207 distinct protein coding mRNAs.

Six genes were also selected for exon level analysis based on literature (Table 2). Isoforms of these genes were previously reported as potentially important in lung cancer. All genes, excluding HLA-G, were alternatively spliced according to both RNA-seq and HTA analyses. However, only RUNX1 had exons and junctions that were repeatedly identified by exon usage and junction usage in both platforms.

**Table 2:** Some alternatively spliced genes involved in lung cancer identified in both platforms

Gene	Ensembl ID	Reference	Sites found by RNA-seq for		Sites found by HTA for		Concordant
			exons	junctions	exons	junctions	
TP63	ENSG00000073282	Lo Iacono et al. [57]	6	10	2	27	No
TP73	ENSG00000078900	Lo Iacono et al. [57]	0	2	2	6	No
CD44	ENSG00000026508	Wang et al. [58]	34	32	19	43	No
HLA-G	ENSG00000204632	Yan et al. [59]	2	0	0	0	No
POSTN	ENSG00000133110	Morra et al. [60]	3	5	1	7	No
RUNX1	ENSG00000159216	Ito et al. [61]	8	2	18	13	Yes

Surprisingly, the reproducibility between the platforms for differential exon and junction analysis was low. In order to investigate this observation in detail we focussed on the potential bias in the location of spliced exons in a gene, their length and GC-content. RNA-seq tended to identify more differentially used exons in the 3′ end of a gene (red line in Fig. 6d), while HTA found more significantly spliced exons in the middle (blue line). This may be mainly linked to the length of the exons as 3′ exons are on average longer. The distributions on Fig. 6e confirm it: the exons identified by RNA-seq were longer compared to the exons detected by HTA. No bias was observed in the GC content of significant exons.

Next, we investigated the linkage between significance of differential exon usage and two potentially linked parameters: average gene expression and differential gene expression. The growth of significantly spliced exons for both platforms are compared in Additional file 1: Figure S10a. As HTA has much smaller dynamic range and thus faster growth, we corrected the numbers by considering quantiles of average gene expression (Additional file 1: Figure S10b). Based on these images, HTA detects more splicing events in lowly and moderately expressed genes, while RNA-seq has a bias towards highly expressed genes. Differential expression of a gene can influence the detection of its differential splicing. We compared intersections between differentially expressed and differentially spliced genes in Additional file 1: Figure S10c,d and observed a higher overlap between lists of differential expressed and spliced genes in HTA data than in RNA-seq.

**Fig. 7:**
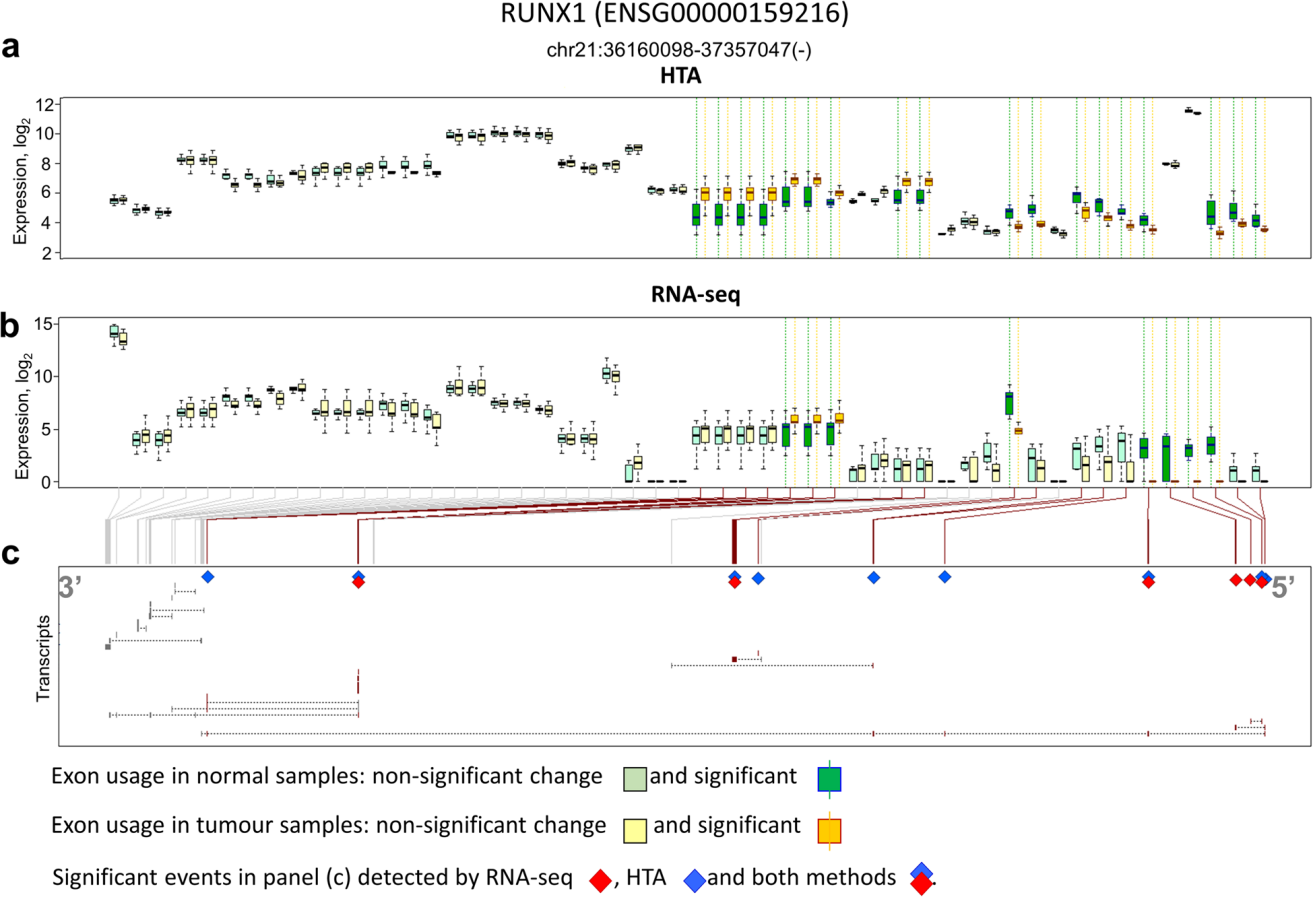
Exon differential usage in the RUNX1 gene. The results from HTA (**a**) and RNA-seq (**b**) are presented. Transcripts of RUNX1 are shown in (**c**) and significant exons are highlighted by diamonds. Exons are presented based on their Ensembl annotation, therefore the same exon can be shown by several equivalent pairs of boxes. RNA-seq shows a higher number of reads at the 3′ end than at the at 5′ end, where the important splicing events are found

We then visually inspected the exon expression profiles and also identified several biases in RNA-seq data. The RUNX1 gene was taken as an example to illustrate the general tendency observed (Fig. 7). RNA-seq reported on average higher expression for exons in the 3′ end (left side of Fig. 7b) and lower expression in the 5′-end (right side of Fig. 7b). In the RUNX1 gene, these exons were essential to identify several short transcripts (Fig. 7c). Differential usage of exons captured by HTA data was more consistent (Fig. 7a).

The discrepancy in exon usage can result from differences in the data or from disagreement of the analysis algorithms. In order to investigate this issue, we applied the same DEA paradigm, which was used for gene expression, directly to the expression of exons. The DEA of exons is much simpler than the analysis of differential usage of exons, as no normalization of gene expression is required. Remarkably, DEA showed strongly improved concordance between the lists of differentially expressed exons for the platforms, with a Jaccard index of 0.44 (the same thresholds for differential exon usage were used: FDR < 0.05, |logFC| > log_2_(1.5)). Thus, the disagreement between the splicing analysis algorithms played a strong role in the observed discrepancy.

We finalized the splicing analysis by functional annotation of the spliced genes (Additional file 1: Figure S11a,b). For each platform, we considered only genes that were consistently found by exon and junction analyses. The results were similar to those from the functional annotation of differentially expressed genes: HTA data revealed more enriched GO terms (Additional file 1: Figure S11a), RNA-seq showed higher preference for the extracellular matrix and HTA for the nuclear compartment. Consistently spliced genes (207) were also functionally annotated and the results are shown in Additional file 1: Figure S11b; they suggest that splicing occurred in genes involved in cytoskeleton formation, cell projection, cilium movement and processes at the extracellular matrix (Additional file 4).

## Discussion

In general, microarray and sequencing showed similar results and a high level of correlation (*r* = 0.76) when measuring the expression of protein coding mRNAs from clinical samples. We observed that GC-correction strongly improved the correlation between HTA and RNA-seq results. The obtained correlation is in the range of previous observations: Raghavachari et al. reported a correlation of 0.64 between RNA-seq and Affymetrix Human Exon 1.0 ST arrays [47]. The value of correlation may be affected by other factors even within one platform. In fact, different biochemical protocols used in RNA-seq technology were found to be responsible for correlation fluctuations between 0.83–0.86 [48]. High correlation between the two platforms was also observed in the analysis of known oncogenes for lung squamous cell carcinoma. Regarding differences between tumour and normal samples, over 50% of significantly differentially expressed genes were equally found by both platforms. The genes found in common were better confirmed by the large reference TCGA dataset than the genes uniquely identified by one platform. Finally, both platforms identified the key differences between tumour and normal samples at functional level. Increased cell division and loss of normal tissue functions, such as cilium activity, were only observed in tumour samples.

Despite the general similarity of the data sets, we repeatedly detected higher variability in sequencing data than in microarray data. Indeed, the fraction of unexplained variability was much higher for RNA-seq, especially for lncRNAs. Biological replicates also showed more variability in sequencing data, notably for the genes that were characterized by a low number of mapped reads. Finally, a higher number of genes were found to be differentially expressed (FDR < 0.01) or to have strong predictive power (AUC > 0.95) discriminating tumour from normal tissue using the microarray platform.

One cause for the high RNA-seq variability is linked to the sensitivity of sequencing to the length of the genes. Many genes with a length below 2000 nt are either missed or underestimated by sequencing. The length factor may explain the strong decrease in correlation between the two platforms (*r* = 0.32) regarding lncRNA expression: the lengths of the considered lncRNAs were substantially lower (medial length ~ 700 nt) than the lengths of protein coding mRNAs (median length ~ 3800 nt). Transcripts of lncRNAs contain less exons (median is 3) than transcripts of mRNAs (median is 5). Another cause for this variability may be linked to gene abundance. When comparing biological replicates, RNA-seq repeatedly showed higher variability, especially for low expressed genes. A similar observation was made by Zhao et al. [17]. This factor also influenced the results of the gene enrichment analysis. HTA identified more genes enriched in biological functions specific to the nucleus while functions uniquely identified by RNA-seq were related to the extracellular matrix. Expression of the genes from the former group was lower than expression of genes from the later. This can be explained by the fact that proteins active in the nucleus are required, on average, in lower quantity than proteins active at the cellular membrane. Thus, mRNA of genes related to the extracellular matrix is expressed at a higher level compared to nucleus related genes. These findings contradict to some extent the conclusions of Zhang et al. [49], who found that microarrays are more sensitive to genes coding for membrane proteins, and sequencing – for genes in the nucleus. However, these authors used a completely different paradigm: they considered housekeeping genes, while we performed the analysis based on differentially expressed ones.

Important characteristics of each platform are the detection limit and the dynamic range. In literature, there is no consensus on which expression level should be considered as a limit of detection in RNA-seq. This threshold value should be related to the quality of the samples and sequencing depth. In terms of reads per kilobase per million (RPKM), a threshold of 0.125 [50] or 0.3 [51] was proposed after analysis of false discovery rate behaviour. Some works suggest setting a threshold on detected counts. McIntyre et al. observed a strong increase of variability in exon expression when the number of counts per exon falls below 5 [52]. In the paper of the SEQC/MAQC-III consortium, genes with more than 16 reads are considered as expressed [53]. In our work, we calculated the detection limit and dynamic range of the platforms using only significantly differentially expressed protein-coding genes as described in Methods. Using this approach, overestimation of the dynamic range was avoided. As expected, higher dynamic range was observed for the RNA-seq approach, nevertheless the microarray was able to detect smaller variations in expression level than RNA-seq. Summarising our observations, it should be concluded that the larger dynamic range of RNA-seq is limited by its larger variability of expression, especially for low abundant transcripts. If there is a need to detect only slight changes in gene expression, especially for low expressed transcripts, the use of microarrays may be advisable.

The final topic to be considered is the platform performance in identification of gene isoforms which may originate from alternative splicing in normal tissue and aberrant splicing in cancer. This task is the most challenging one in transcriptomics studies and can be prone to high variability of outcomes and lack of accuracy [5, 54]. The problem has so far been considered by many authors; some claim that transcript reconstruction should be performed in the form of deconvolution of mixed transcripts rather than considering individual exons [55], others oppose this statement as the task of deconvolution is inherently difficult, and its outcome strongly dependent on the completeness of transcript annotation and questionable in terms of accuracy and robustness [56]. We selected the second approach and aimed at the detection of exons and exon junctions that are differentially used in transcripts produced by tumour and normal cells. Estimating the expression of exons with RNA-seq data, we first tried to use the same counting method for exon expression that we used for genes – *HTSeq*. However, a very low correlation between the platforms for exon expression was observed – around 0.2 (the correspondent correlation at gene level was 0.76). This discrepancy was caused by two facts: first, the human genome contains many overlapping exons which are considered as different entities in the Ensembl annotation; second, the size of many exons is comparable to the size of a read (77 bases in our case), and thus the same read is shared between two exons via exon-exon junctions. These facts make a large portion of sequencing reads ambiguous – belonging to several exons. Such reads are omitted by *HTSeq* counting, as this tool is not designed for exon quantification. Therefore, in order to count exons in a reasonable way we used the recently presented method of *Rsubread* package of R/Bioconductor, which resulted in an increase of the average Spearman correlation to 0.66. Despite the high correlation for exon expression, the results of differential exon/junction usage analysis were very discordant. Even within one platform the similarity index between the lists of differentially used exons (originated from exon and junction analyses) was low: the best concordance was observed for HTA (Jaccard index of 0.16). Similarity between the two platforms varied between 0.03–0.08. Once again, two reasons may be responsible for this discordance, at least partially: dependency of RNA-seq on the length of gene/exon and lack of reads mapped on low abundant exons. Regarding the length, RNA-seq expression of shorter exons is not correlated with HTA-based expression, contrary to the expression of long exons. In addition, we observed a strong bias for splicing detection towards the 3′ end and an increased detection of alternative splicing for long exons in RNA-seq. These two facts are linked, as the last 3′ terminal exons are usually longer than other exons in the transcripts, they drag more reads and therefore their expression has a higher signal to noise ratio. Finally, a large number of the spliced exons that were found significant from RNA-seq data originated from highly expressed genes. At the same time, HTA detected more differentially expressed genes among those that were differentially spliced, which can be an artefact of the analysis method.

The second issue results from the low exon coverage, which is not enough for accurate quantification of exon abundance. While microarrays can work with low quantities of RNA, a large library size is needed for RNA-seq in order to estimate the expression of short and low abundant regions. In our experiments, 43% of the genes had less than 100 reads on average, which was enough to estimate gene expression, but produced highly variable results at exon level.

Both methods were able to identify splicing events in a small selected group of genes which were linked to cancer at isoform level in literature [57–61]. However, only the RUNX1 gene was found spliced at the same point by both platforms. Nevertheless, in our view, the limited set of alternatively used exons, which were detected by both platforms, are quite interesting. They originate from 207 genes which are strong candidates for further biological investigation, as they undergo alternative or aberrant splicing in lung squamous cell carcinoma. These genes are linked mainly to the cytoskeleton and extracellular matrix.

Finally, we determined that the methods of alternative splicing analysis were at least partially responsible for the discordant results obtained. Simple DEA at exon level showed much higher concordance, similar to DEA at gene level (Jaccard index of 0.44), between the platforms. A recent report from Dapas et al. [62] shows that DEA results show high similarity at the transcript level as well. In addition, the lists of genes with differential splicing usage were more similar between the platforms than the lists of differentially used exons. This may be caused by a tendency of certain genes (for example – differentially expressed) to be found differentially spliced more often.

This observation supports the statement that analysis of differential exon usage based only on RNA-seq data may be inadvisable [4, 5]. We recommend prudence interpreting the results of alternative splicing. Intersection of the outcomes from several platforms and additional validation are required when identifying alternatively used exons.

## Conclusions

Evaluated expressions of protein-coding genes were consistent in HTA and RNA-seq platforms: expression patterns were highly correlated, differentially expressed gene lists were similar and relevant biological processes were successfully identified. At the same time, RNA-seq always showed higher stochastic variability of the results compared to HTA arrays. This mainly originated from an insufficient number of reads from short and low abundant genes.

Both RNA-seq and HTA can be applied for detection of non-coding lncRNAs, however larger libraries are needed to quantify lncRNAs properly, as their length and abundance are lower than for protein coding mRNAs.

Analysis of alternative splicing or, more specifically, differential exon usage, produced discordant results between the platforms and even within the same platform. We expect that applying platforms independently will generate a high level of false positive detection and only by combining microarray and sequencing results true splicing events may be identified. Alternatively, these results should undergo experimental validation.

Based on our considerations, when researchers need to compare relatively large groups of samples and are aiming at known genes, they should rather choose microarray techniques, which will provide them with fast, cheap and concordant results. Contrary, for thorough analysis of a small number of samples, especially when unknown transcripts should be discovered, the only reasonable option would be the deep sequencing approach.

## Additional files

Additional file 1: Supplementary Figures. (PPTX 1864 kb)

Additional file 2: The lists of significantly differentially expressed coding genes and lncRNAs identified by HTA and RNA-seq platforms. (XLSX 2698 kb)

Additional file 3: Significantly enriched GO terms. (XLSX 125 kb)

Additional file 4: The list of consistent exons that were detected by both platforms and functional annotations of the corresponding genes. (XLSX 104 kb)

## Abbreviations

AUC: Area under ROC curve
CPM: Counts per million
DEA: Differential expression analysis
FDR: False discovery rate
FPKM: Fragments per kilobase of transcript per million mapped reads
GG-H: GLUE Grant human transcriptome arrays
GO: Gene ontology
HTA: Affymetrix® Human Transcriptome Arrays 2.0
lncRNA: Long non-coding RNA
logFC: Log_2_ fold change
miRNA: Micro RNA
mRNA: Messenger RNA
PCA: Principal component analysis
PVCA: Principal variance component analysis
RIN: RNA integrity number
RLE: Relative log expression
RMA: Robust multi-chip analysis
RNA-seq: RNA sequencing
ROC: Receiver operating characteristic
rRNA: Ribosomal RNA
SCC: Squamous cell carcinoma
SI: Splicing index
SNP: Single nucleotide polymorphism
TCGA: The Cancer Genome Atlas
TMM: Weighted trimmed mean method
