# Supplementary material for: RNA sequencing and transcriptome arrays analyses show opposing results for alternative splicing in patient derived samples
Source: BMC Genomics. 2017 Jun 6;18:443. doi: 10.1186/s12864-017-3819-y (PMC5461714; doi:10.1186/s12864-017-3819-y)
Supplement: Supplementary file 1 — Supplementary Figures. (PPTX 1864 kb) [file 12864_2017_3819_MOESM1_ESM.pptx]

## Slide 1
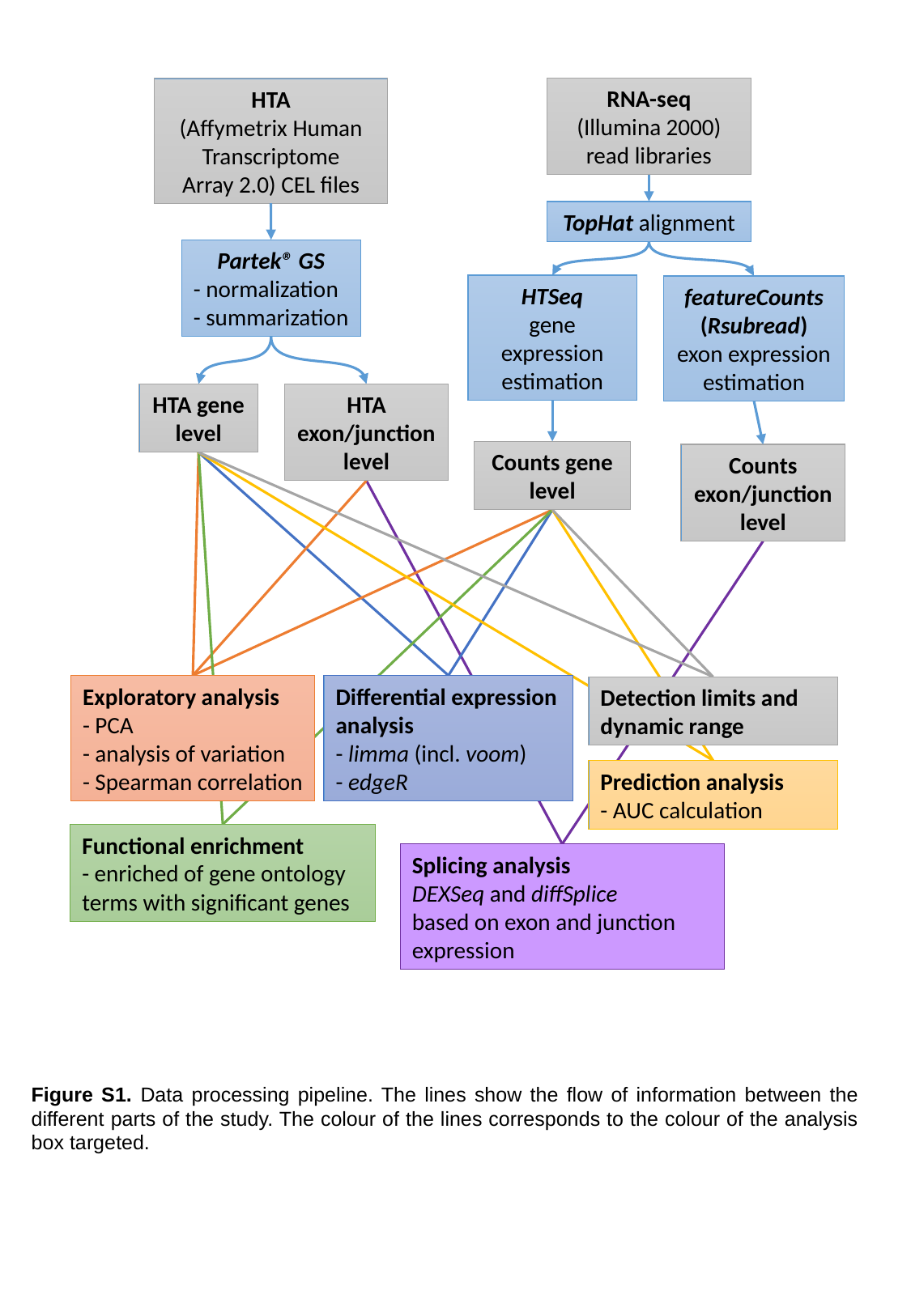

RNA-seq
(Illumina 2000) read libraries
HTA
(Affymetrix Human Transcriptome
Array 2.0) CEL files
TopHat alignment
Partek® GS
- normalization
- summarization
HTSeq
gene expression estimation
featureCounts
(Rsubread)
exon expression estimation
HTA gene level
HTA exon/junction level
Counts gene level
Counts exon/junction level
Exploratory analysis
- PCA
- analysis of variation
- Spearman correlation
Differential expression analysis
- limma (incl. voom)
- edgeR
Detection limits and dynamic range
Prediction analysis
- AUC calculation
Functional enrichment
- enriched of gene ontology terms with significant genes
Splicing analysis
DEXSeq and diffSplice
based on exon and junction expression
Figure S1. Data processing pipeline. The lines show the flow of information between the different parts of the study. The colour of the lines corresponds to the colour of the analysis box targeted.

## Slide 2
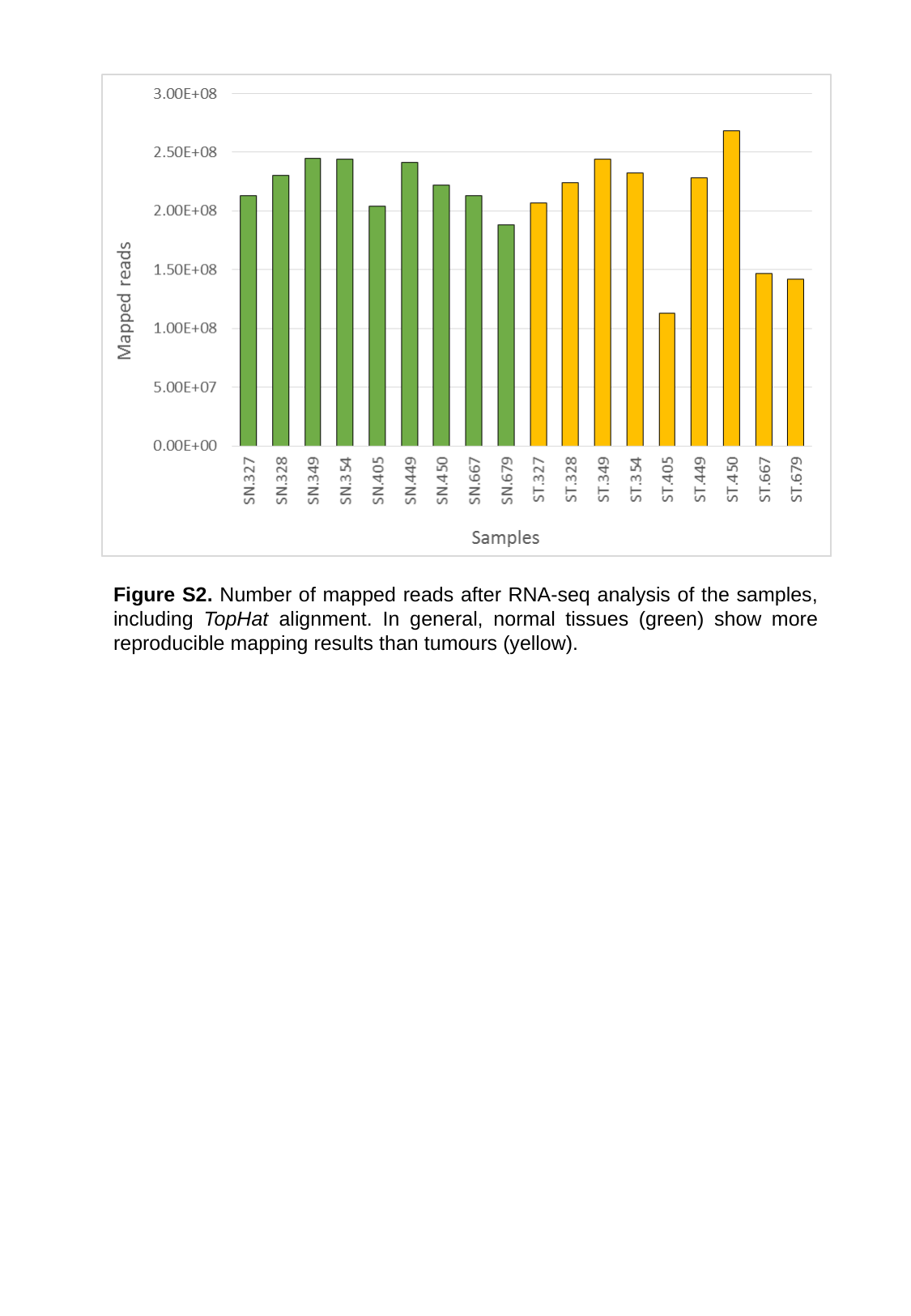

Figure S2. Number of mapped reads after RNA-seq analysis of the samples, including TopHat alignment. In general, normal tissues (green) show more reproducible mapping results than tumours (yellow).

## Slide 3
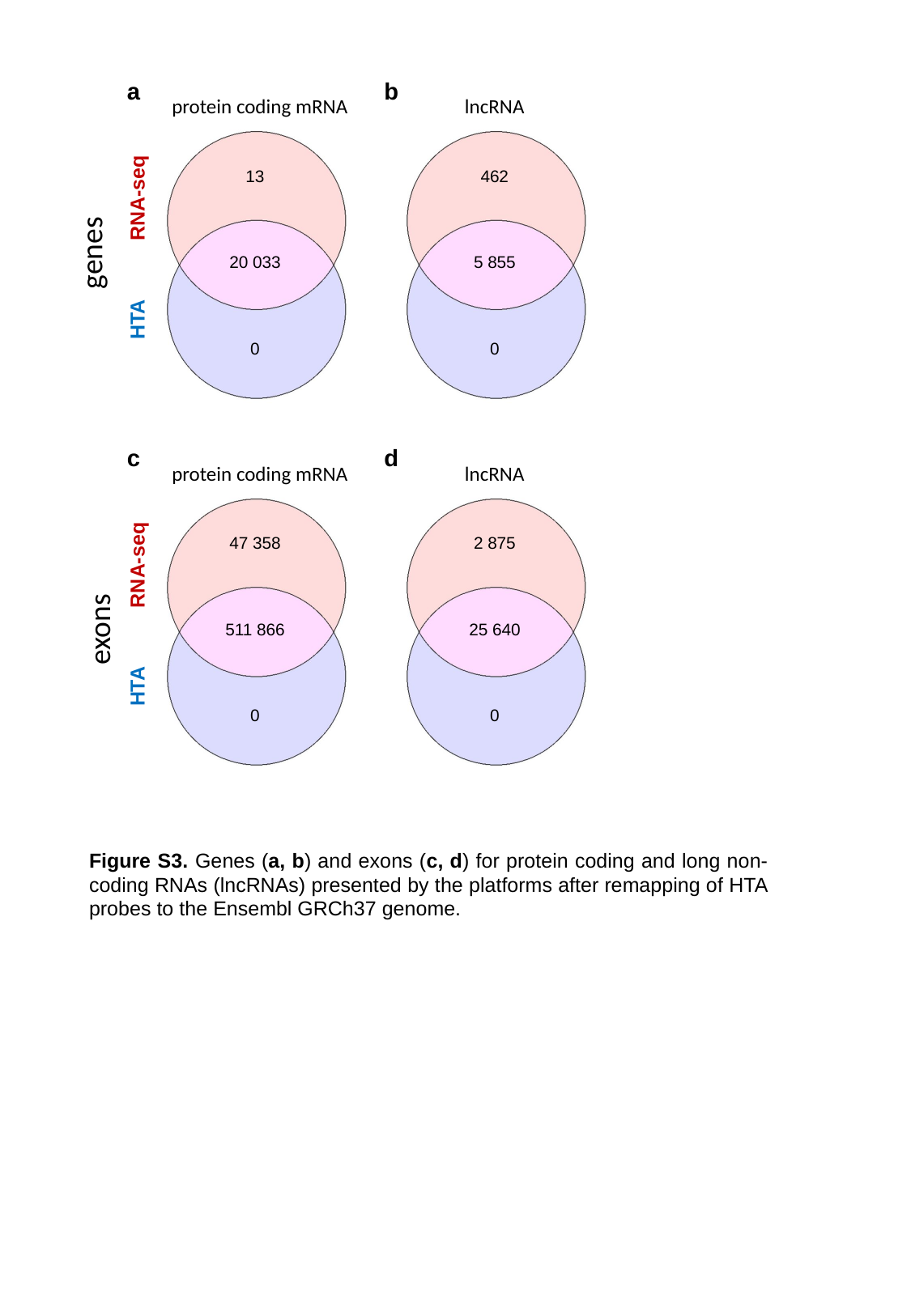

a
b
protein coding mRNA
13
20 033
0
lncRNA
462
5 855
0
RNA-seq
genes
HTA
c
d
protein coding mRNA
47 358
511 866
0
lncRNA
2 875
25 640
0
RNA-seq
exons
HTA
Figure S3. Genes (a, b) and exons (c, d) for protein coding and long non-coding RNAs (lncRNAs) presented by the platforms after remapping of HTA probes to the Ensembl GRCh37 genome.

## Slide 4
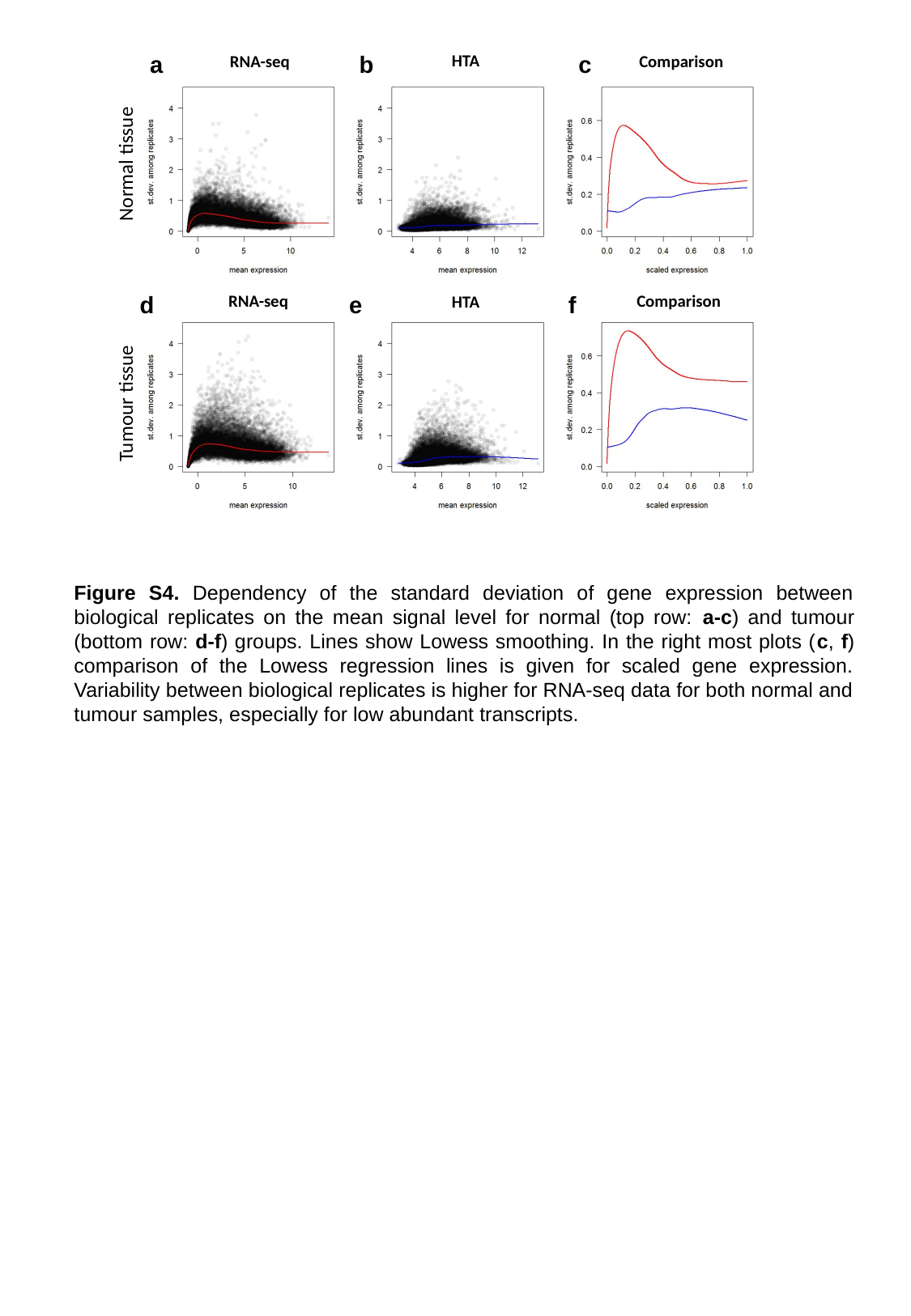

b
HTA
c
a
RNA-seq
Comparison
Normal tissue
e
f
d
Comparison
RNA-seq
HTA
Tumour tissue
Figure S4. Dependency of the standard deviation of gene expression between biological replicates on the mean signal level for normal (top row: a-c) and tumour (bottom row: d-f) groups. Lines show Lowess smoothing. In the right most plots (c, f) comparison of the Lowess regression lines is given for scaled gene expression. Variability between biological replicates is higher for RNA-seq data for both normal and tumour samples, especially for low abundant transcripts.

## Slide 5
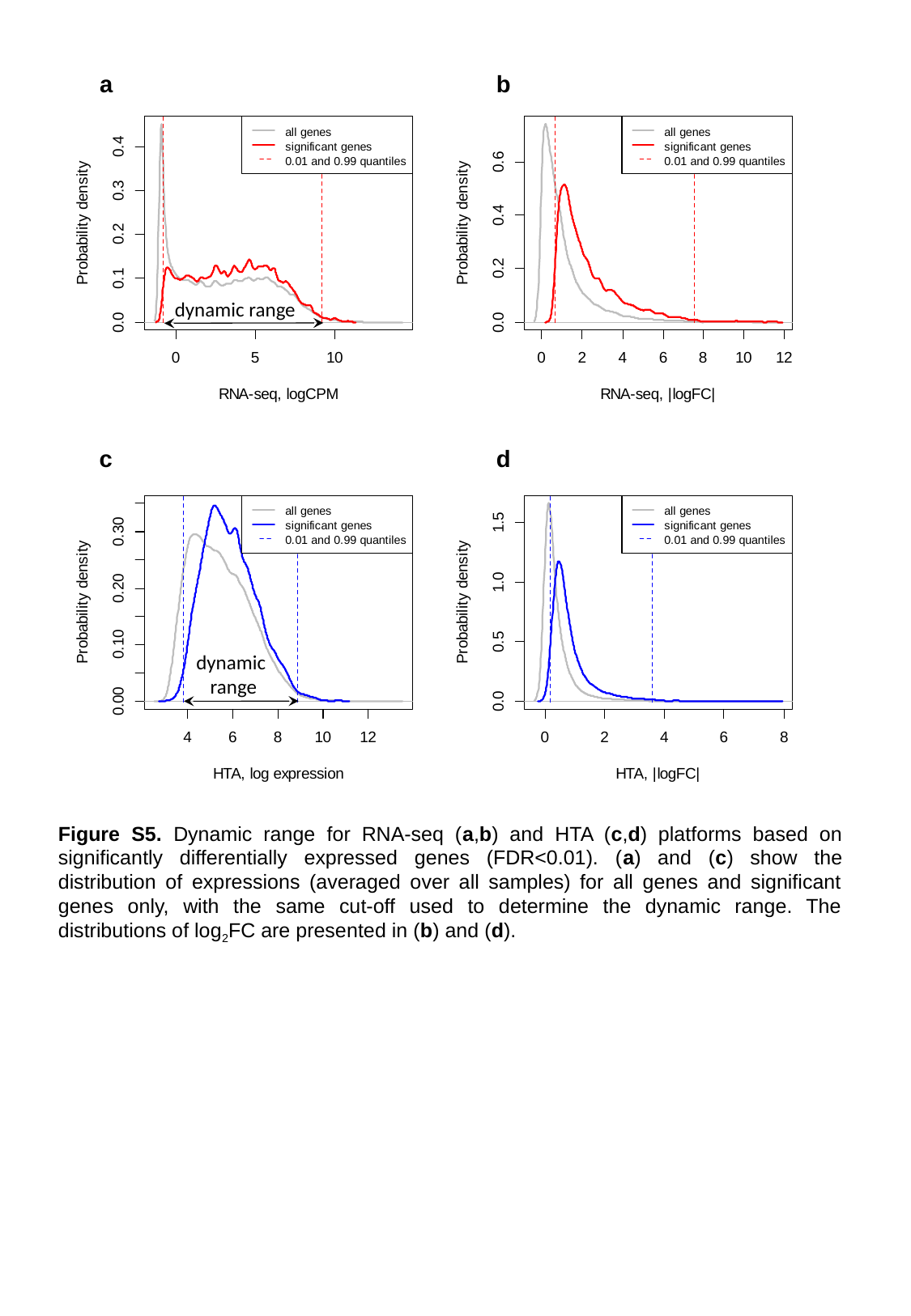

a
b
dynamic range
c
d
dynamic
 range
Figure S5. Dynamic range for RNA-seq (a,b) and HTA (c,d) platforms based on significantly differentially expressed genes (FDR<0.01). (a) and (c) show the distribution of expressions (averaged over all samples) for all genes and significant genes only, with the same cut-off used to determine the dynamic range. The distributions of log2FC are presented in (b) and (d).

## Slide 6
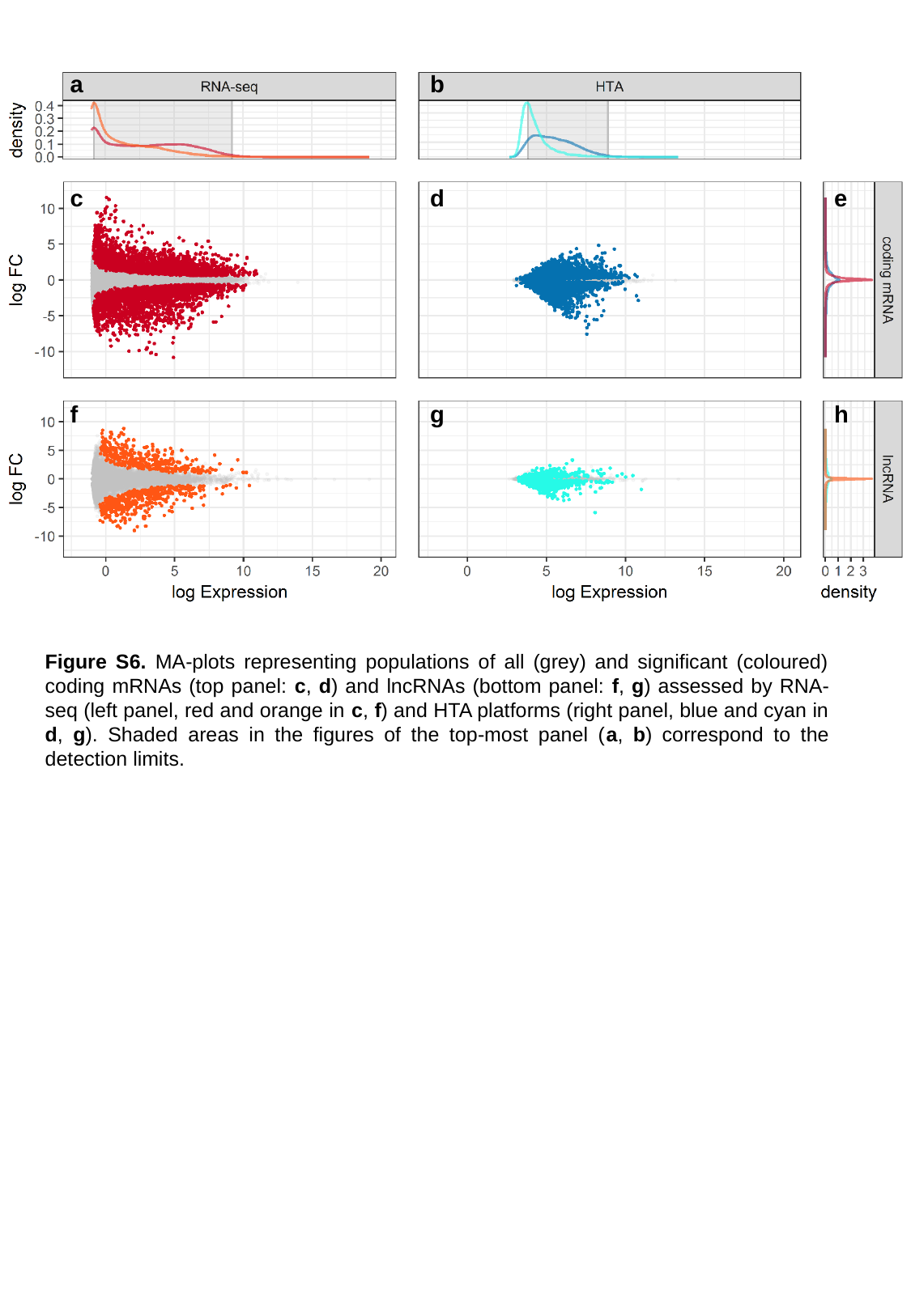

a
b
e
c
d
h
g
f
Figure S6. MA-plots representing populations of all (grey) and significant (coloured) coding mRNAs (top panel: c, d) and lncRNAs (bottom panel: f, g) assessed by RNA-seq (left panel, red and orange in c, f) and HTA platforms (right panel, blue and cyan in d, g). Shaded areas in the figures of the top-most panel (a, b) correspond to the detection limits.

## Slide 7
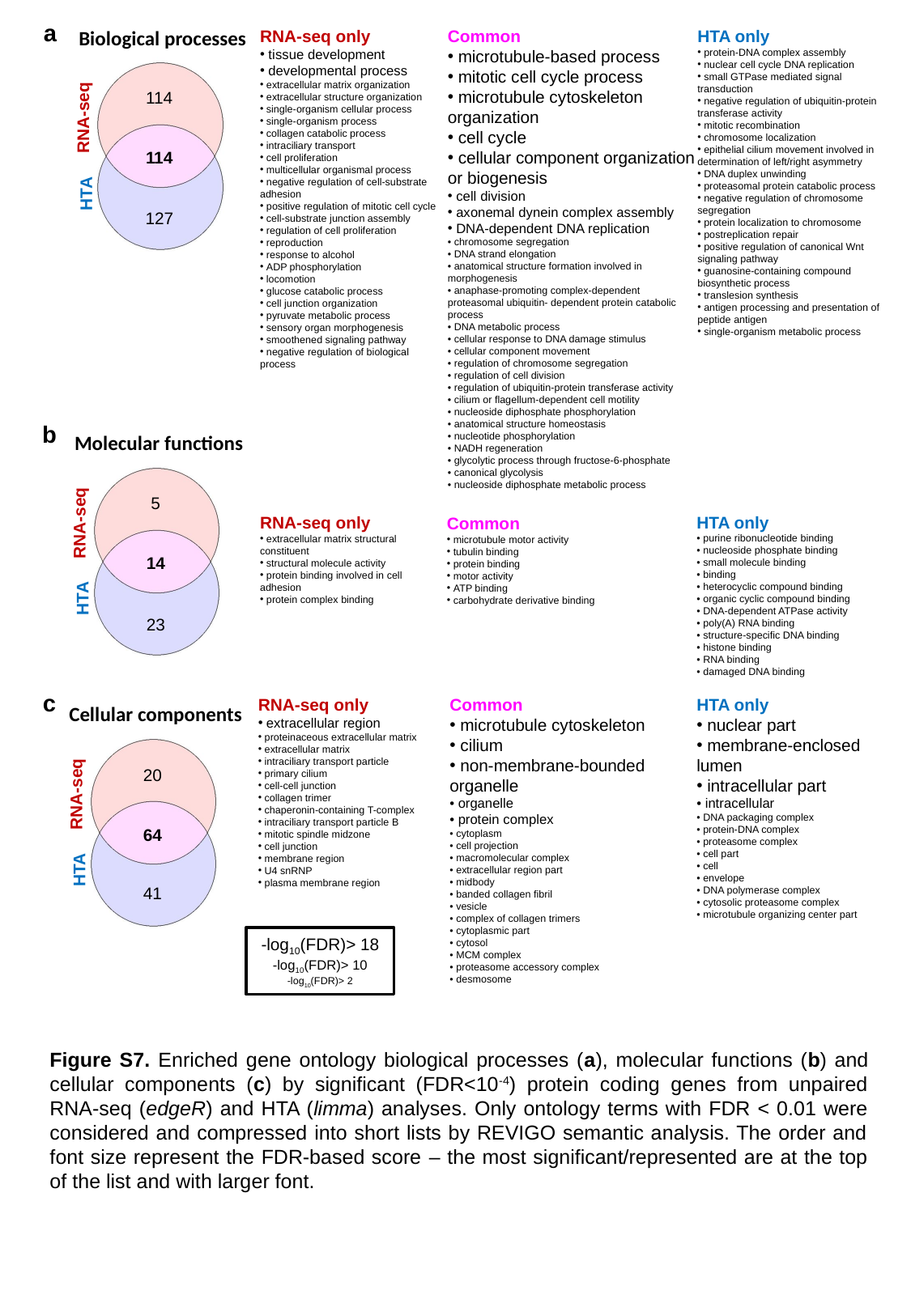

a
Biological processes
114
RNA-seq
114
HTA
127
RNA-seq only
 tissue development
 developmental process
 extracellular matrix organization
 extracellular structure organization
 single-organism cellular process
 single-organism process
 collagen catabolic process
 intraciliary transport
 cell proliferation
 multicellular organismal process
 negative regulation of cell-substrate adhesion
 positive regulation of mitotic cell cycle
 cell-substrate junction assembly
 regulation of cell proliferation
 reproduction
 response to alcohol
 ADP phosphorylation
 locomotion
 glucose catabolic process
 cell junction organization
 pyruvate metabolic process
 sensory organ morphogenesis
 smoothened signaling pathway
 negative regulation of biological process
Common
 microtubule-based process
 mitotic cell cycle process
 microtubule cytoskeleton organization
 cell cycle
 cellular component organization or biogenesis
 cell division
 axonemal dynein complex assembly
 DNA-dependent DNA replication
 chromosome segregation
 DNA strand elongation
 anatomical structure formation involved in morphogenesis
 anaphase-promoting complex-dependent proteasomal ubiquitin- dependent protein catabolic process
 DNA metabolic process
 cellular response to DNA damage stimulus
 cellular component movement
 regulation of chromosome segregation
 regulation of cell division
 regulation of ubiquitin-protein transferase activity
 cilium or flagellum-dependent cell motility
 nucleoside diphosphate phosphorylation
 anatomical structure homeostasis
 nucleotide phosphorylation
 NADH regeneration
 glycolytic process through fructose-6-phosphate
 canonical glycolysis
 nucleoside diphosphate metabolic process
HTA only
 protein-DNA complex assembly
 nuclear cell cycle DNA replication
 small GTPase mediated signal transduction
 negative regulation of ubiquitin-protein transferase activity
 mitotic recombination
 chromosome localization
 epithelial cilium movement involved in determination of left/right asymmetry
 DNA duplex unwinding
 proteasomal protein catabolic process
 negative regulation of chromosome segregation
 protein localization to chromosome
 postreplication repair
 positive regulation of canonical Wnt signaling pathway
 guanosine-containing compound biosynthetic process
 translesion synthesis
 antigen processing and presentation of peptide antigen
 single-organism metabolic process
b
Molecular functions
5
RNA-seq
14
HTA
23
HTA only
 purine ribonucleotide binding
 nucleoside phosphate binding
 small molecule binding
 binding
 heterocyclic compound binding
 organic cyclic compound binding
 DNA-dependent ATPase activity
 poly(A) RNA binding
 structure-specific DNA binding
 histone binding
 RNA binding
 damaged DNA binding
RNA-seq only
 extracellular matrix structural constituent
 structural molecule activity
 protein binding involved in cell adhesion
 protein complex binding
Common
 microtubule motor activity
 tubulin binding
 protein binding
 motor activity
 ATP binding
 carbohydrate derivative binding
c
Cellular components
20
RNA-seq
64
HTA
41
RNA-seq only
 extracellular region
 proteinaceous extracellular matrix
 extracellular matrix
 intraciliary transport particle
 primary cilium
 cell-cell junction
 collagen trimer
 chaperonin-containing T-complex
 intraciliary transport particle B
 mitotic spindle midzone
 cell junction
 membrane region
 U4 snRNP
 plasma membrane region
Common
 microtubule cytoskeleton
 cilium
 non-membrane-bounded organelle
 organelle
 protein complex
 cytoplasm
 cell projection
 macromolecular complex
 extracellular region part
 midbody
 banded collagen fibril
 vesicle
 complex of collagen trimers
 cytoplasmic part
 cytosol
 MCM complex
 proteasome accessory complex
 desmosome
HTA only
 nuclear part
 membrane-enclosed lumen
 intracellular part
 intracellular
 DNA packaging complex
 protein-DNA complex
 proteasome complex
 cell part
 cell
 envelope
 DNA polymerase complex
 cytosolic proteasome complex
 microtubule organizing center part
-log10(FDR)> 18
-log10(FDR)> 10
-log10(FDR)> 2
Figure S7. Enriched gene ontology biological processes (a), molecular functions (b) and cellular components (c) by significant (FDR<10-4) protein coding genes from unpaired RNA-seq (edgeR) and HTA (limma) analyses. Only ontology terms with FDR < 0.01 were considered and compressed into short lists by REVIGO semantic analysis. The order and font size represent the FDR-based score – the most significant/represented are at the top of the list and with larger font.

## Slide 8
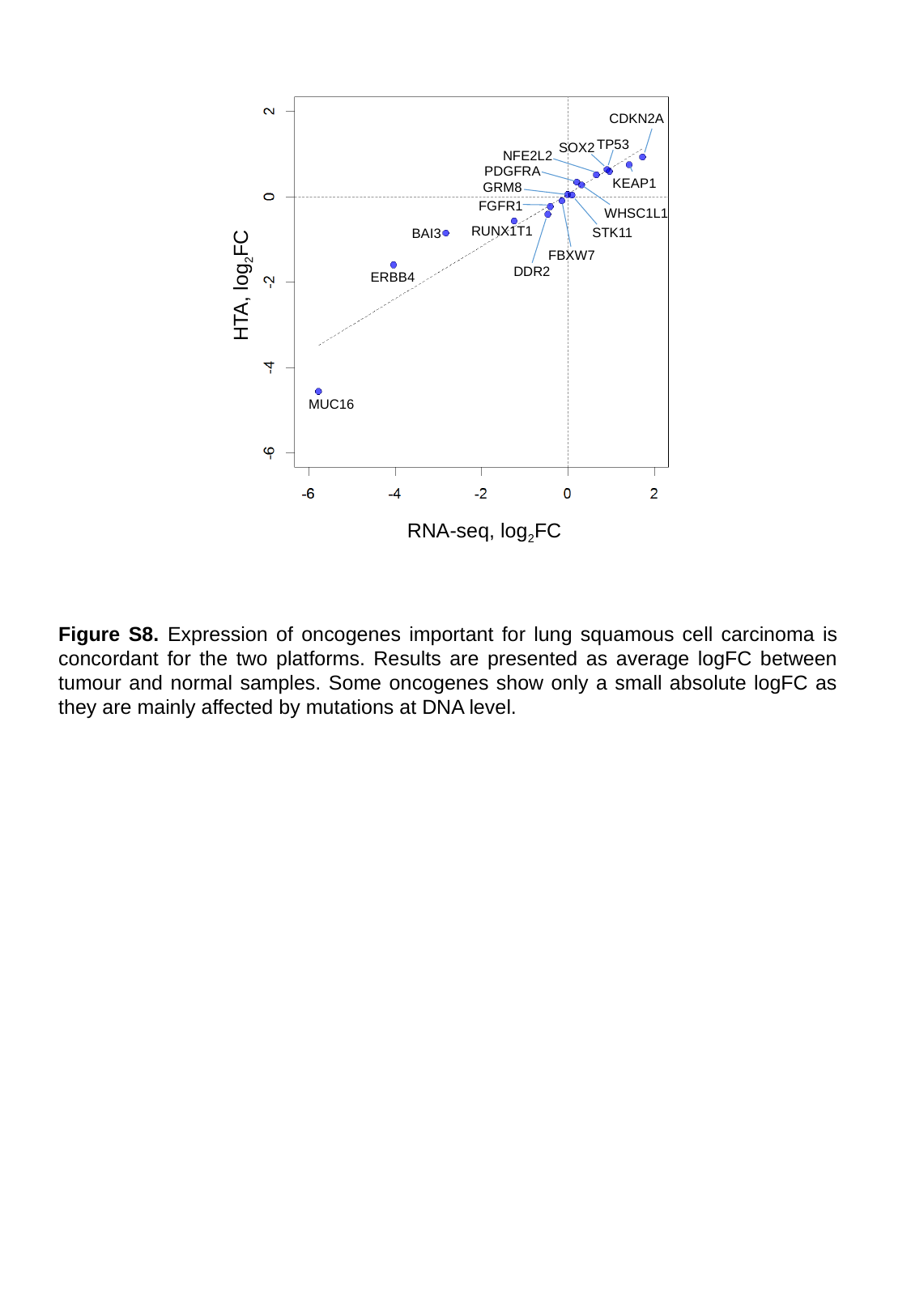

CDKN2A
TP53
SOX2
NFE2L2
PDGFRA
KEAP1
GRM8
FGFR1
WHSC1L1
RUNX1T1
STK11
BAI3
FBXW7
DDR2
ERBB4
HTA, log2FC
MUC16
RNA-seq, log2FC
Figure S8. Expression of oncogenes important for lung squamous cell carcinoma is concordant for the two platforms. Results are presented as average logFC between tumour and normal samples. Some oncogenes show only a small absolute logFC as they are mainly affected by mutations at DNA level.

## Slide 9
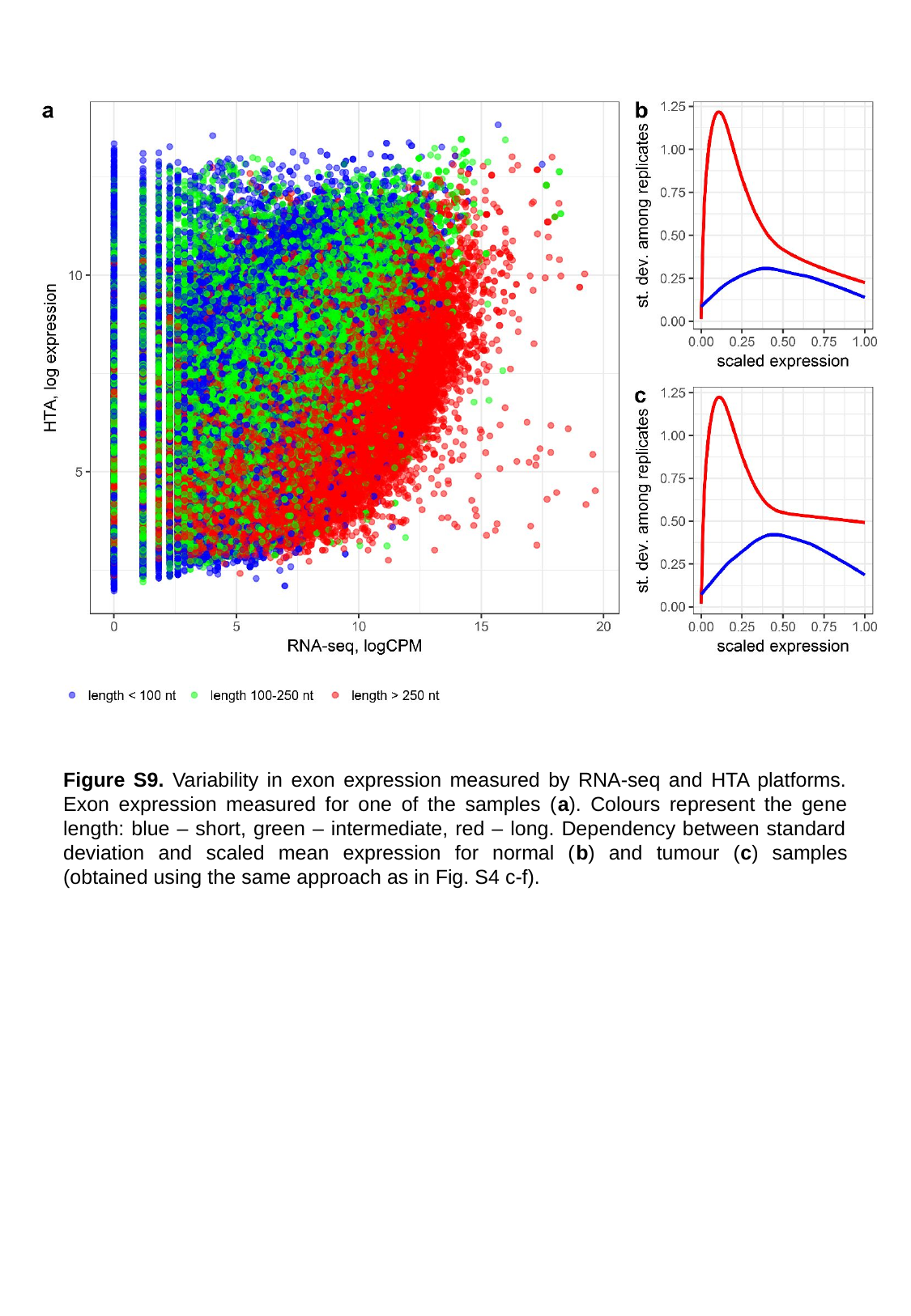

Figure S9. Variability in exon expression measured by RNA-seq and HTA platforms. Exon expression measured for one of the samples (a). Colours represent the gene length: blue – short, green – intermediate, red – long. Dependency between standard deviation and scaled mean expression for normal (b) and tumour (c) samples (obtained using the same approach as in Fig. S4 c-f).

## Slide 10
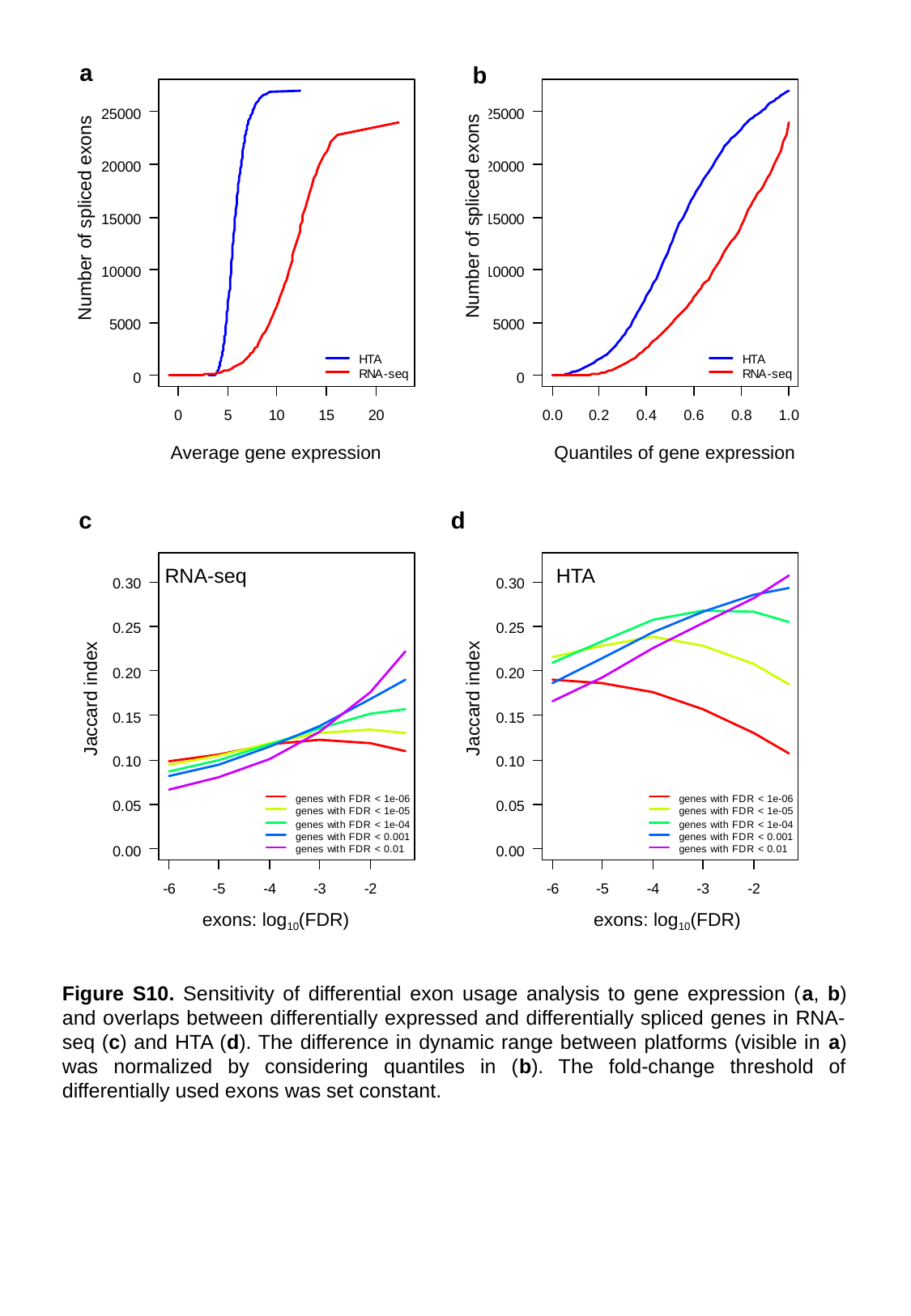

a
b
Number of spliced exons
Number of spliced exons
Average gene expression
Quantiles of gene expression
c
d
RNA-seq
HTA
Jaccard index
Jaccard index
exons: log10(FDR)
exons: log10(FDR)
Figure S10. Sensitivity of differential exon usage analysis to gene expression (a, b) and overlaps between differentially expressed and differentially spliced genes in RNA-seq (c) and HTA (d). The difference in dynamic range between platforms (visible in a) was normalized by considering quantiles in (b). The fold-change threshold of differentially used exons was set constant.

## Slide 11
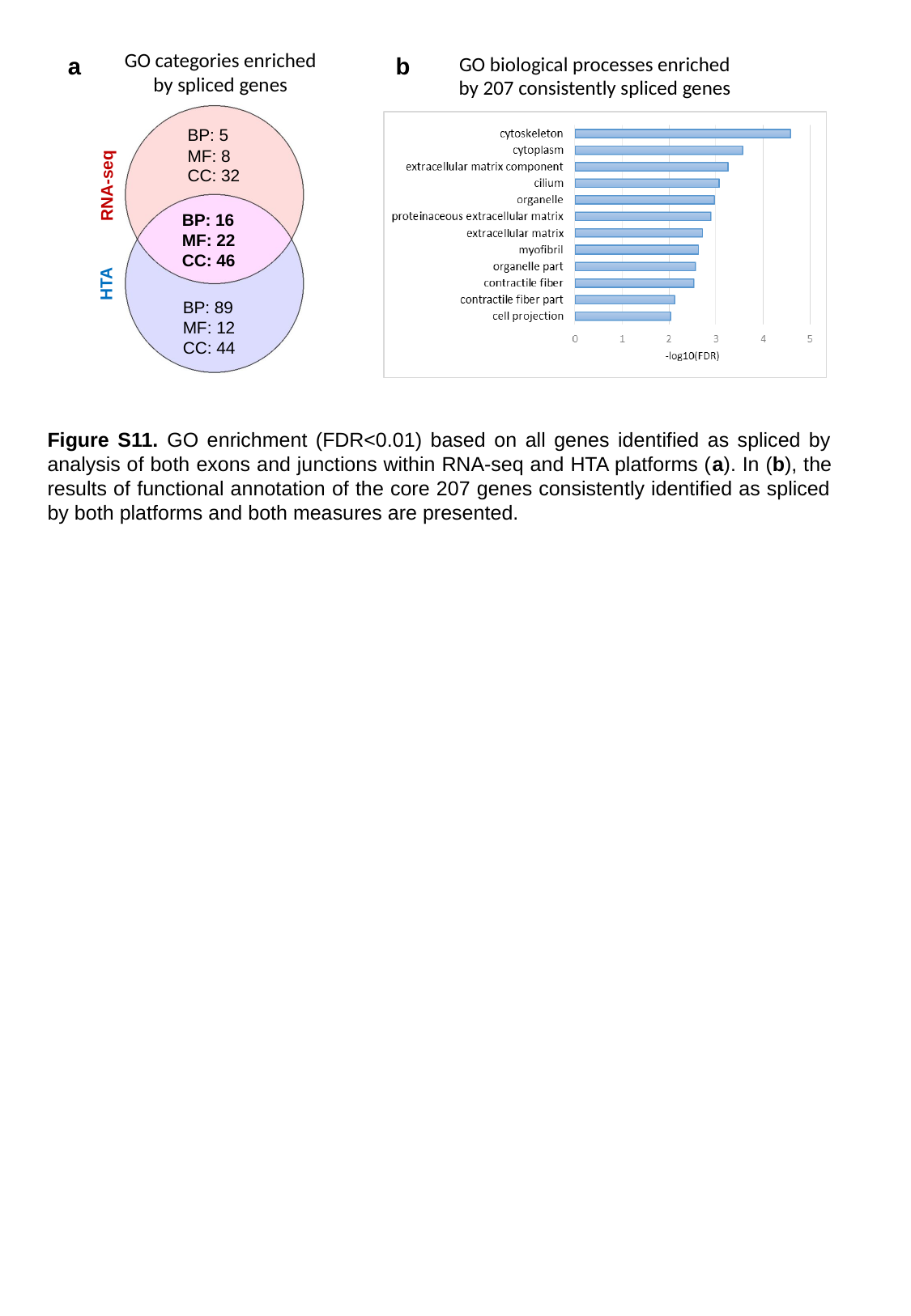

GO categories enriched
by spliced genes
BP: 5
MF: 8
CC: 32
BP: 16
MF: 22
CC: 46
BP: 89
MF: 12
CC: 44
GO biological processes enriched
by 207 consistently spliced genes
a
b
RNA-seq
HTA
Figure S11. GO enrichment (FDR<0.01) based on all genes identified as spliced by analysis of both exons and junctions within RNA-seq and HTA platforms (a). In (b), the results of functional annotation of the core 207 genes consistently identified as spliced by both platforms and both measures are presented.
